# Population genomics of a predatory mammal reveals patterns of decline and impacts of exposure to toxic toads

**DOI:** 10.1111/mec.16680

**Published:** 2022-09-25

**Authors:** Brenton von Takach, Louis Ranjard, Christopher P. Burridge, Skye F. Cameron, Teigan Cremona, Mark D. B. Eldridge, Diana O. Fisher, Stephen Frankenberg, Brydie M. Hill, Rosemary Hohnen, Chris J. Jolly, Ella Kelly, Anna J. MacDonald, Adnan Moussalli, Kym Ottewell, Ben L. Phillips, Ian J. Radford, Peter B. S. Spencer, Gavin J. Trewella, Linette S. Umbrello, Sam C. Banks

**Affiliations:** ^1^ Research Institute for the Environment and Livelihoods Charles Darwin University Darwin Northern Territory Australia; ^2^ School of Molecular and Life Sciences Curtin University Perth Western Australia Australia; ^3^ The Research School of Biology, Faculty of Science The Australian National University Acton Australian Capital Territory Australia; ^4^ PlantTech Research Institute Tauranga New Zealand; ^5^ School of Natural Sciences University of Tasmania Hobart Tasmania Australia; ^6^ Australian Wildlife Conservancy Kimberley Western Australia Australia; ^7^ School of Biological Sciences University of Queensland St Lucia Queensland Australia; ^8^ Australian Museum Research Institute Sydney New South Wales Australia; ^9^ School of BioSciences University of Melbourne Parkville Victoria Australia; ^10^ Flora and Fauna Division, Department of Environment, Parks and Water Security Northern Territory Government Northern Territory Australia; ^11^ Institute of Land, Water and Society, School of Environmental Science Charles Sturt University Albury New South Wales Australia; ^12^ School of Natural Sciences Macquarie University Macquarie Park New South Wales Australia; ^13^ Australian Antarctic Division, Department of Agriculture Water and the Environment Kingston Tasmania Australia; ^14^ Department of Science Museums Victoria Melbourne Victoria Australia; ^15^ Department of Biodiversity, Conservation and Attractions Perth Western Australia Australia; ^16^ Environmental and Conservation Sciences, Murdoch University Perth Western Australia Australia; ^17^ Collections and Research Centre Western Australian Museum Welshpool Western Australia Australia

**Keywords:** biogeography, cane toad, demographic history, genomic diversity, northern quoll, population structure

## Abstract

Mammal declines across northern Australia are one of the major biodiversity loss events occurring globally. There has been no regional assessment of the implications of these species declines for genomic diversity. To address this, we conducted a species‐wide assessment of genomic diversity in the northern quoll (*Dasyurus hallucatus*), an Endangered marsupial carnivore. We used next generation sequencing methods to genotype 10,191 single nucleotide polymorphisms (SNPs) in 352 individuals from across a 3220‐km length of the continent, investigating patterns of population genomic structure and diversity, and identifying loci showing signals of putative selection. We found strong heterogeneity in the distribution of genomic diversity across the continent, characterized by (i) biogeographical barriers driving hierarchical population structure through long‐term isolation, and (ii) severe reductions in diversity resulting from population declines, exacerbated by the spread of introduced toxic cane toads (*Rhinella marina*). These results warn of a large ongoing loss of genomic diversity and associated adaptive capacity as mammals decline across northern Australia. Encouragingly, populations of the northern quoll established on toad‐free islands by translocations appear to have maintained most of the initial genomic diversity after 16 years. By mapping patterns of genomic diversity within and among populations, and investigating these patterns in the context of population declines, we can provide conservation managers with data critical to informed decision‐making. This includes the identification of populations that are candidates for genetic management, the importance of remnant island and insurance/translocated populations for the conservation of genetic diversity, and the characterization of putative evolutionarily significant units.

## 1 INTRODUCTION

Declines of vertebrate species are occurring globally due to habitat degradation, invasive species, land clearing and harvesting (Díaz et al., 2019). The Australian continent and surrounding islands harbour highly unique faunas and have been exceptionally vulnerable to such changes, with 33 mammal species becoming extinct since European colonization in 1788 (Roycroft et al., 2021; Woinarski et al., 2019). As species continue to decline, population genomic analysis provides an opportunity to identify historical patterns of gene flow and population structure, as well as investigate and quantify the impacts of decline on genetic diversity. With genetic diversity a critically important component of global biodiversity measures (Hoban et al., 2021), such analyses can benefit threatened species management by providing data on populations that are candidates for genetic intervention, quantifying the importance of remnant island and insurance/translocated populations for conserving genetic diversity, or characterizing putative evolutionarily significant units (Frankham et al., 2017; Ralls et al., 2018; von Takach, Penton, et al., 2021; Wright et al., 2020). Broadly, consideration of these factors can assist in the conservation of adaptive potential of threatened populations, by promoting genetic diversity and adaptive variation (Kelly & Phillips, 2016; Madsen et al., 1999; von Takach Dukai et al., 2019).

The invasion of exotic species into new environments, and the associated novel selection pressures that invasive species impose, can induce a range of adaptive evolutionary responses in native species (Berthon, 2015; Strauss et al., 2006). Importantly, when substantial declines of native populations occur, the resulting loss of standing genomic variation within populations is likely to hinder adaptation to subsequent environmental change (i.e., loss of adaptive potential). Few studies have quantified the impacts of invasive species on population genomic diversity on declining native species, with most relevant studies investigating the genetic consequences of disease outbreaks (Schoville et al., 2011; Serieys et al., 2015).

One species that is well suited for such analysis is the northern quoll (*Dasyurus hallucatus*). The northern quoll is a small (<1500 g) generalist marsupial predator (Oakwood, 1997) that was, until relatively recently, abundant across a broad distribution and range of ecosystems in northern Australia (Oakwood, 2008), and spans several biogeographical breaks that drive the population genetic structure of many taxa (Bowman et al., 2010; Catullo et al., 2014; Edwards et al., 2017; Eldridge et al., 2012; Rollins et al., 2012). Since European colonization of Australia, from 1788 onwards, the northern quoll has declined across much of its former distribution (Moore et al., 2019), partly due to pernicious and compounding threats such as land clearing, inappropriate fire and grazing regimes, and the impact of feral cats (Braithwaite & Griffiths, 1994). From 1935 onwards, the decline of the northern quoll has been greatly accelerated by the introduction and continuing spread of cane toads (*Rhinella marina*) in Australia (Burnett, 1997; Shine, 2010).

The cane toad is a lethally toxic amphibian introduced into northeastern Australia in a failed attempt at biological control of a pest of the sugar cane industry—the cane beetle (Shine et al., 2020). Although the introduction of cane toads failed to impede the impacts of cane beetles, cane toads flourished in this novel setting, and have since colonized much of northern Australia (Doody et al., 2019; Phillips et al., 2003; Sabath et al., 1981). Like many Australian taxa, the northern quoll has no evolutionary history of exposure to toad (Bufonidae) toxins (cf. rodents; Cabrera‐Guzmán et al., 2015), and are therefore extremely sensitive to the toxic defences of cane toads (Phillips et al., 2003; Smith & Phillips, 2006). As with some large Australian predators (e.g., freshwater crocodiles *Crocodylus johnstoni*; Letnic et al., 2008), northern quolls mistake cane toads for suitable prey and suffer precipitous population‐level impacts upon their arrival (Shine, 2010). Consequently, northern quolls have been extirpated from many localities (Burnett, 1997; Moore et al., 2019; Oakwood & Foster, 2008; Woinarski et al., 2010; Woinarski, Legge, et al., 2011).

The northern quoll is now listed as Endangered under state, national and international listings (IUCN Red List), and populations are being actively managed by various jurisdictions. Because the spread of cane toads has so far proved impossible to halt, current management actions are aimed at mitigating the impact of toads. For example, the Northern Territory government established insurance populations on two offshore islands in 2003 (Rankmore et al., 2008), and there is ongoing research into the feasibility of increasing the frequency of toad‐avoidance traits in populations via training (Indigo et al., 2021; Jolly et al., 2020) or translocation of heritable “toad‐smart” genotypes into toad‐naïve populations (“targeted gene flow”) (Kelly et al., 2021; Kelly & Phillips, 2019). Critically, these management strategies require understanding and managing various genetic aspects for them to be successfully implemented.

Here, we provide a comprehensive assessment of the population genomic structure of the northern quoll, illustrating how patterns of genomic diversity may be impacted by cane toad invasion and various management strategies, to improve the efficacy of decision‐making. By assembling one of the most comprehensive sets of samples of any Australian mammal species to date, including toad‐naïve, toad‐exposed, island and translocated populations, we investigate patterns of genomic diversity and population genomic structure within and among these treatments, as well as demonstrate impacts of the ongoing spread of cane toads. We also identify putatively adaptive loci that have higher‐than‐expected levels of differentiation (suggestive of diversifying selection) as well as loci that are significantly associated with the presence of cane toads. We predict that our genomic analyses will identify (i) population genomic structure resulting from vicariance across biogeographical barriers; (ii) patterns of drift associated with isolated island populations and (iii) large impacts of cane toads, or other drivers of population decline, on population genomic diversity, with population bottlenecks in toad‐exposed populations potentially contributing to a reduction in genetic diversity. Our temporal replication will also enable us to directly observe the effect of establishing insurance populations on offshore islands in the short term (16 years). We suggest that this study will provide a quantitative example of the impacts that invasive species can have on the patterns of genomic diversity in native species, the conclusions of which will benefit attempts to mitigate the impacts of invasive species.

## 2 MATERIALS AND METHODS

### 2.1 Sample collection

**FIGURE 1:**
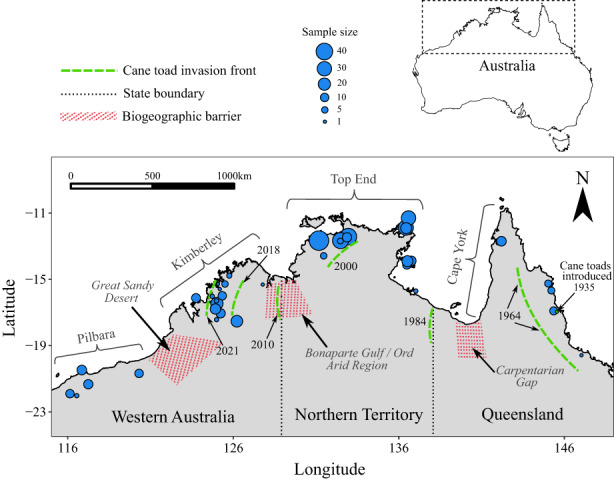
Locations (blue points) of northern quoll tissue samples collected for population genomic analyses. Sampling points are sized relative to the number of individuals sampled. The spread of cane toads across northern Australia is depicted by green lines indicating the arrival of the invasion front at various dates. Approximate locations of major known biogeographical barriers in northern Australia are also depicted

We collated tissue samples from across the entire species distribution from multiple personnel who had been involved in mammal trapping between 1993 and 2019, covering the regional jurisdictions of Queensland (QLD), the Northern Territory (NT) and Western Australia (WA) (Figure 1). While some localities had all samples collected in a single year, others were sampled during multiple visits across one or two years, with low capture rates making large sample sizes in any one session difficult to obtain. Trapping efforts within a locality were variable among jurisdictions and field teams, but typically involved sampling a set of several small grids (e.g., 1 ha) of cage traps spread over 10–20 km. All samples taken from the set of grids within a locality were given an identifying name for population genomic analysis. In total, 459 samples were collected from 41 localities, with five localities in QLD, 17 localities in the NT and 19 localities in WA (Figure S1). Four of the localities were trapped on separate occasions, which we have treated independently (Table S1). Of these 46 independent sampling periods/localities, 21 were represented by 10 or more individuals and 30 were represented by six or more individuals. The mean pairwise geographical distance between all sampled localities was 883 km, with a maximum distance between sampled individuals of 3220 km.

For each sampling locality, we determined whether cane toads were present at the date of collection by cross‐referencing with published timelines of cane toad spread (Brown et al., 2015; Doody et al., 2019; Phillips et al., 2007; Urban et al., 2008). Localities were identified as belonging to one of four treatments, including (i) those that had never been exposed to cane toads (“toad‐naïve”); (ii) those that have been exposed to cane toads (“toad‐exposed”); (iii) those naturally occurring on toad‐free islands (“island”); and (iv) those established via translocations onto toad‐free islands (“translocated”). Islands were considered separately from the toad‐naïve treatment because long periods of isolation and small effective population sizes at these locations have generally resulted in low levels of genetic diversity (Cardoso et al., 2009; Spencer et al., 2017). Translocated populations of northern quolls on Astell Island and Pobassoo Island have been present since 2003, when the species was introduced from the NT mainland to the islands as insurance populations (Cardoso et al., 2009). In total, 24 of the 46 independent sampling periods/locations were classed as toad‐naïve, 10 were classed as toad‐exposed, and eight were from naturally toad‐free islands (NT and WA only). One location in the NT, “East Alligator,” had >10 samples collected both prior to, and after, the arrival of cane toads to the region, allowing for preliminary before‐and‐after comparisons at this locality to be made.

### 2.2 DNA extraction and sequencing

Ear tissue samples (~3 mm^2^) were prepared and extracted in plate format (Qiagen DNeasy 96 Blood & Tissue Kit) following the standard protocol with an extended lysis (incubation at 56°C for 2 h then reduced to 37°C overnight). Samples consisting of dried blood spots on filter paper were extracted with a slight modification to the lysis step (Choi et al., 2014), where ~25 mm^2^ of filter paper containing dried blood was incubated at 37°C overnight in 400 μl of 1× PBS buffer, prior to completion of the Qiagen protocol from the addition of ethanol at step three.

Double‐stranded DNA concentrations were quantified using a Qubit 3.0 Fluorometer and normalized to 200 ng DNA in 25 μl, and then arranged on 96‐well plates, where each plate contained two within‐plate technical replicates, one technical replicate from each of the other plates and one negative control (blank) well. Plates were sent for double‐digest restriction‐associated DNA (ddRAD) sequencing at the Australian Genome Research Facility in Melbourne, Victoria. An optimal combination of two restriction enzymes was determined using three establishment samples (broadly representative of the species distribution), with *Pst*I and *Nla*III considered most suitable for achieving the best level of amplification and minimizing repetitive sequences. Briefly, the library preparation protocol consisted of (i) digestion using *Pst*I and *Nla*III, (ii) ligation with one of 48 unique inline barcoded adapters compatible with the restriction site overhang, (iii) manual sample pooling, (iv) DNA purification (QIAquick PCR Purification Kit followed by SPRIselect paramagnetic beads), (v) size‐selection targeting fragments of 280–375 bp in size (BluePippin, Sage Science) and (vi) a PCR (polymerase chain reaction) amplification step where one of two multiplexing index primers was added. Indexed libraries were pooled together and loaded onto flow cells for 150‐bp single‐end sequencing on either an Illumina NextSeq 500 (three plates) or a NovaSeq 6000 (two plates) platform.

### 2.3 Bioinformatic pipeline and SNP filtering

Raw sequence data were demultiplexed using the *process_radtags* function of stacks (Catchen et al., 2013), with checking for intact RAD sites and reads truncated to 135 bp to meet a quality threshold of 30. Of the 1.647 billion total reads, 95.8% were retained after demultiplexing. These were then mapped to the draft assembly of the northern quoll reference genome (scaffold N50 89 Mb) (S. Frankenberg, A. Moussalli, and B.L. Phillips unpub.) using the bwa version 0.7.17 *mem* algorithm (Li, 2013), outputting a sequence/alignment map (SAM) file for each sample. Each SAM file was then compressed to a binary alignment map (BAM) file with samtools version 1.7‐1 (Li et al., 2009) used to (i) filter out unmapped reads; (ii) sort reads by chromosome and position and (iii) index each file.

The program angsd version 0.93 (Korneliussen et al., 2014) was used to filter reads and call single nucleotide polymorphisms (SNPs) to output an SNP‐by‐sample matrix. Reads were only used to call SNPs if the mapping quality score was ≥20 (thus excluding reads that mapped poorly or mapped to repeat regions of the genome). Loci were only retained if they (i) were polymorphic, as inferred by a likelihood ratio test *p* value ≤1 × 10^−5^ (Kim et al., 2011); (ii) were genotyped in at least 50% of individuals; (iii) had a minimum of five reads per locus per sample and (iv) had fewer than 150 reads per locus per sample (removing bias due to paralogues or repetitive elements). Genotypes were called with a posterior probability threshold of at least 0.98 (using GATK genotype likelihoods) using a uniform prior. This resulted in the identification of 402,328 SNPs across 475 samples with a mean read depth per SNP per individual of 13 and a median read depth per SNP per sample of 12. This SNP‐by‐sample matrix was read into the R version 4.0.3 statistical programming package (R Core Team, 2020) for further SNP filtering and all subsequent analyses.

In R, we filtered SNPs based on (i) the level of missing data; (ii) observed heterozygosity; (iii) minor allele counts and (iv) whether they showed signals of being under selection. We filtered individuals based on (i) the level of missing data and (ii) relatedness to other genotyped individuals within sampled localities. To filter SNPs and samples based on the level of missing data, we used a custom script to iteratively remove individuals and SNPs with decreasing missing data thresholds until no SNP had more than 3% missing data and no individual had more than 20% missing data. We then removed any SNP with a minor allele count <3 (O'Leary et al., 2018) or an observed heterozygosity (*H*_O_) >0.6 (excluding 50 SNPs with potentially erroneously merged reads), and then pruned SNPs based on linkage disequilibrium via the “SNPRelate” package (Zheng et al., 2012), with a maximum of 500,000 bp in the sliding window and a correlation coefficient threshold of .5. This resulted in the retention of 10,676 SNPs and 368 unique individuals (with an additional 15 technical replicates still present).

To ensure that relationships between individuals could be accurately inferred from the data, we used these SNPs and samples to construct a hierarchical clustering dendrogram based on genetic distance, with visual examination of the dendrogram confirming that all technical replicates paired closely together on long branches (Figure S2). We also made a higher‐level bootstrapped dendrogram by using genetic distances among sampling localities instead of individuals (Figure S3). The percentage difference between called genotypes of technical replicates was also used to confirm that genotyping error rates were low (mean 99.99% ± 0.003% *SE* similarity between replicates). One of each pair was then removed from all further analyses. The data set was then further pruned to remove highly related individuals and SNPs showing signals of putative selection, both of which can bias analyses that were developed using the assumption of unrelated individuals and neutrally evolving loci.

To estimate relatedness between individuals, we used the recently developed method‐of‐moments estimate for the kinship coefficient (Goudet et al., 2018; Weir & Goudet, 2017), implemented in the “hierfstat” package (Goudet, 2005). This approach estimates kinship values between pairs of individuals relative to the average kinship values of all pairs of individuals in the sampled population (i.e., within each locality). This avoids the problematic requirement for estimation of reference allele frequencies. We found 23 pairs of highly related individuals (kinship coefficients >0.25, i.e., full sib or higher) in a total of six sampling localities, and randomly removed one of each pair, retaining a total of 352 samples for use in the remaining analyses.

### 2.4 Putatively adaptive loci

We investigated the SNP panel for genetic signatures of local adaptation using two methods: identification of SNPs with higher‐than‐expected levels of differentiation (*F*_ST_ outliers), and identification of SNPs that were significantly associated with the presence of cane toads (via a latent factor mixed model). To identify outlier SNPs that showed signatures of diversifying selection, we first computed an ancestry matrix for each individual using the methods implemented in the LEA package (Frichot & François, 2015) and calculated an *F*_ST_ statistic based on this ancestry matrix (Caye et al., 2016; Martins et al., 2016). The *F*_ST_ statistic was then transformed into squared *z*‐scores, with *p*‐values computed using a chi‐squared distribution (Frichot & François, 2015; Weir, 1996). The Benjamini–Hochberg algorithm (Benjamini & Hochberg, 1995), with a false discovery rate of 1 in 1000, was used to correct for issues associated with multiple testing. To identify SNPs associated with the presence of cane toads, we first imputed all missing genotype data, via the “impute” function of the LEA package (method = “mode”), based on the ancestry matrix computed above. As with many genotype–environment association methods, latent factor mixed models require no missing data. The estimated length of time (number of years) that each population had been exposed to cane toads was used as the environmental predictor variable with which genotypes were associated. We generated latent factor mixed models (via the “lfmm2” exact least‐squares function of the LEA package) to identify allele frequencies that were correlated with cane toad occurrence (Caye et al., 2019; Frichot & François, 2015). This method controls for population structure via a number of latent factors equal to the number of ancestral populations (von Takach, Ahrens, et al., 2021). The *p*‐values for each SNP were adjusted using the in‐built robust estimate of the genomic inflation factor to improve the shape of the histogram (Martins et al., 2016) and subjected to a Benjamini–Hochberg algorithmic correction (Benjamini & Hochberg, 1995) to ensure a low rate of false discovery (1 in 1000).

We recorded the genomic locations of all SNPs under putative selection, investigated the extent of overlap in results between the two methods, and then removed all SNPs (485 in total) from the data set that were identified using either method. This produced a selectively neutral data set for population genomic analyses, many of which rely on assumptions of neutrality. While future work may attempt to further elucidate and understand the patterns and genomic architecture of selection in response to cane toad invasion, the set of candidate loci identified here (Table S4) provides a starting point for understanding adaptation across the northern quoll genome.

After all filtering steps had been conducted, we retained a total of 10,191 SNPs and 352 individuals for the remaining analyses. The overall level of missing data for the final filtered SNP‐by‐individual matrix was 1.2%.

### 2.5 Population genomic diversity and structure

Using the filtered SNP data set, we calculated mean values of standard genomic parameters for each sampling locality within each treatment, including the number of alleles (*A*), effective number of alleles (*A*_E_), observed heterozygosity (*H*_O_), expected heterozygosity (*H*_E_), Wright's inbreeding coefficient (*F*_IS_) and the locus polymorphic index (*P*_E_). The East Alligator site was separated into toad‐naïve (2003) and toad‐exposed (2014 and 2017) subsets prior to calculations being made. Similarly, the historical (2005) samples from Astell Island were separated from the more recent (2016) samples. Calculations were made using the “gstudio” package (Dyer, 2016) with the standard small sample size correction used to account for bias in estimates of expected heterozygosity resulting from differences in sample size between localities. As heterozygosity calculations based on filtered SNP data can potentially show biases due to sample sizes and filtering parameters, we also calculated observed and expected values of autosomal heterozygosity for each locality using the methods of Schmidt et al. (2021), which considers both monomorphic and polymorphic nucleotides. This included building aligned sequences into a stacks catalogue via the “ref_map” pipeline, with the filtered and sorted BAM files as inputs (using only previously filtered individuals), and analysing the data set using the core program “Populations” with all missing sites removed. The heterozygosity estimates and standard errors in the subsection of the summary output titled “# All positions (variant and fixed)” were recorded, and Pearson's product moment correlation coefficient was used to determine the strength of the relationship between values of autosomal heterozygosity and SNP heterozygosity.

We visualized geographical patterns of population genomic structure using a principal coordinate plot of pairwise genetic distances between individuals. The first two principal coordinate dimensions of the genetic distance matrix were calculated using the *cmdscale* function, and individuals plotted on the coordinates. To investigate patterns of within‐region population structure, we also performed independent principal coordinate analyses on subsets of individuals that formed the major clusters in the comprehensive plot.

To assign individuals to ancestral population genomic clusters, investigate patterns of admixture between populations and assess hierarchical population structure, we used the alternating projected least squares algorithm implemented in the “tess3r” package (Caye et al., 2016, 2018). This method applies a model of genetic structure featuring a discrete number (*k*) of ancestral populations, allowing for independent investigation of values for *k* that have low cross‐entropy metrics (Frichot et al., 2014; Frichot & François, 2015). It also incorporates the spatial location of sampling, to remove bias associated with patterns of isolation‐by‐distance. Cross‐entropy criteria were calculated for values of *k* between 1 and 15, and a cross‐entropy scree‐plot was output for visual identification of a clear plateau or change in curvature in the plot. The matrices of individual admixture coefficients for the most relevant values of *k* for inference were then extracted and plotted as stacked bar plots to visualize hierarchical population structure. To visualize this structure with respect to the geographical location of populations, we interpolated high‐level values of *k* (2–5) across the landscape. To prevent interpolation of genomic structure beyond the geographical range of the northern quoll, we limited the interpolation surface to the known historical (pre‐European) distribution of the species.

To quantify the level of genomic differentiation between localities, we calculated two differentiation metrics for all pairwise comparisons between localities. These included the classic Weir and Cockerham (1984) measure of *F*_ST_ and the standardized differentiation measure $G_{\mathrm{ST}}^{\prime \prime }$. The *F*_ST_ values and their significance levels were calculated using the stamppFst function of the “StAMPP” package (Pembleton et al., 2013), which calculates significance values by bootstrapping across loci (“nboot = 1000”). The $G_{\mathrm{ST}}^{\prime \prime }$ values were calculated using the *pairwise_Gst_Hedrick* function of the “mmod” package (Winter, 2012). The $G_{\mathrm{ST}}^{\prime \prime }$ metric is an *F*_ST_ analogue that uses bias‐corrected estimates of within‐population gene diversity (*H*_S_) and total gene diversity (*H*_T_) to improve inference of demographic history among populations (Hedrick, 2005; Meirmans & Hedrick, 2011). To test the level of correlation between the *F*_ST_ and $G_{\mathrm{ST}}^{\prime \prime }$ metrics, we conducted a Mantel test on the pairwise matrices, using 10,000 permutations.

To visualize patterns of isolation‐by‐distance we constructed a scatterplot of standardized *F*_ST_ values (Rousset, 1997) against geographical distance for all pairwise comparisons of mainland localities (where *n* ≥ 6). A Mantel test was used to test the significance of geographical distance on standardized genomic differentiation, using 10,000 permutations. Isolation‐by‐distance among all mainland individuals was also investigated using spatial autocorrelation of multilocus genotypes, plotted on a correlogram. Pairwise geographical distances between individuals were calculated from latitude and longitude observations for each individual via the *earth.dist* function of the “fossil” package (Vavrek, 2011). Correlation and significance values were made using the *genetic_autocorrelation* function of the “gstudio” package (Dyer, 2016) with 999 permutations, and the results plotted in R.

### 2.6 Demographic and temporal trends

To test the relative influence of vicariance and cane toads on genomic diversity among our natural treatments (toad‐naïve, toad‐exposed and island localities), we constructed four generalized least square models using the “nlme” package (Pinheiro et al., 2020). The response variables were *A*_E_, *F*_IS_, *H*_E_ and *P*_E_, with treatment as the predictor variable, and a Gaussian spatial correlation structure included to account for spatial autocorrelation of predictor values within geographical regions. We note that toad‐exposed populations cluster towards the eastern side of the species distribution, due to the invasion front spreading from the far east. Tukey multiple comparison tests were then used to identify whether mean values for each response were significantly different among treatments. To test the effect that isolation on islands (i.e., Astell Island) or toad invasion and establishment (i.e., East Alligator) had on genomic diversity metrics, we constructed individual generalized linear models for each metric, where loci were samples and the predictor variable was a factor corresponding to the year of the survey.

To identify patterns of historical demography at a locality, including the potential influence of cane toads on population size, we investigated past changes in effective population size (*N*_*e*_) using three methods, of which one is appropriate for estimating recent (i.e., tends to hundreds of generations) trends and two that are most useful for deeper time (i.e., thousands to hundreds of thousands of generations) trends.

To estimate recent historical trends in effective population size (*N*_*e*_), we used the version 1.1 software tool (Barbato et al., 2015), which analyses SNP data based on the relationship between linkage disequilibrium (LD) and *N*_*e*_, with corrections for sample size and recombination rate. A variant call format (VCF) file output from angsd was split into individual VCF files for each sampling locality, with plink version 1.9 (Purcell et al., 2007) used to generate input files for *snep* . We used Sved and Feldman's (1973) mutation rate modifier for correcting the recombination rate, a sample size correction for unphased genotypes, and a recombination rate of 9.1 × 10^−9^ (Feigin et al., 2018). Effective population sizes for the past 200 generations (or to the limit calculated by *snep* ) were plotted and the *x*‐axis scaled to time assuming a mean generation time of 2 years (Pacifici et al., 2013). To increase the available data for the toad‐exposed QLD localities, we combined the localities of Hope Vale and Black Mountain, as these were geographically close and clustered together in the principal coordinates plot. For historical context, we added the timing of the first major European colonization event on the Australian continent (1788). For populations that had been exposed to cane toads at the time of sampling, we also added the timing of cane toad invasion to the locality.

To investigate deeper‐time demographic history, we used two independent techniques: one (the “stairway plot”) that estimates demographic histories based on the composite likelihood of a population's folded site frequency spectrum (Liu & Fu, 2015, 2020), and one (the “extended Bayesian skyline plot” [EBSP]) that uses coalescent theory to trace multilocus lineages backwards through time (Heled & Drummond, 2008).

To build stairway plots for each locality (where *n* ≥ 8), we constructed a folded site frequency spectrum using genotype likelihoods estimated by the angsd software package. All filtering criteria based on mapping and read qualities were the same as previously described, with the filters for polymorphic loci removed to capture information on all monomorphic and polymorphic nucleic sites. By using likelihoods, the site frequency spectrum could be built from between 121 million and 565 million nucleic sites per locality. The site frequency spectrum was loaded into the folded blueprint file provided with the software, with an appropriate mutation rate per site per generation (Feigin et al., 2018), and 200 input files (cf. bootstraps) used to produce the median trend and 95% confidence intervals. The results for each locality were read into R and all plotted on identical scales, with the approximate timing of the last glacial maximum (19,000 years before present) added to each panel for historical context.

We constructed extended Bayesian skyline plots for a subset of localities, to compare results with the stairway plot method and assess their feasibility for use with ddRAD data. We followed the methods of Trucchi et al. (2014), selecting a small number of variable RAD loci (via a custom R script) each 135 bp long, and converting these to NEXUS format for analysis with beast (Suchard et al., 2018). We were able to identify 10 loci for all localities except Weipa, for which only nine loci were found. To minimize bias due to linkage between RAD loci, the selected loci were typically located on separate scaffolds of the reference genome (Table S2), with the minimum distance between any pair of selected loci >11 million base pairs. As in Trucchi et al. (2014), the number of parameters in the model was limited to achieve convergence of the chains. One site model, HKY with four Gamma categories, was defined for each locus class, containing 2–7 SNPs (Table S3). As no information was available to calibrate the substitution rate, we plotted unscaled reconstructions. The Markov chain Monte Carlo (MCMC) analyses, with lengths of 15–40 billion iterations, were run in duplicate for each population to check for adequate convergence. Convergence was checked using tracer (Rambaut et al., 2018) and consistency in estimates of the posterior distribution of the effective sample size. The posterior distributions of the number of population size changes (“sum(indicators.alltrees)” posterior mean) and 95% highest posterior density interval were also recorded to identify whether the constant population size hypothesis could be rejected for each locality.

## 3 RESULTS

### 3.1 Putatively adaptive loci

Together, our outlier and latent factor mixed model analyses identified 485 unique candidate SNPs showing signals of selection, located on 64 genomic scaffolds. Of these 485 SNPs, 259 were identified by outlier analysis, 363 were identified by latent factor mixed model analysis and 137 were identified by both methods. The number of candidate SNPs varied from one to 45 per scaffold (Table S4), with 19 SNPs physically located <100 bp from another candidate SNP.

### 3.2 Population genomic diversity and structure

**FIGURE 2:**
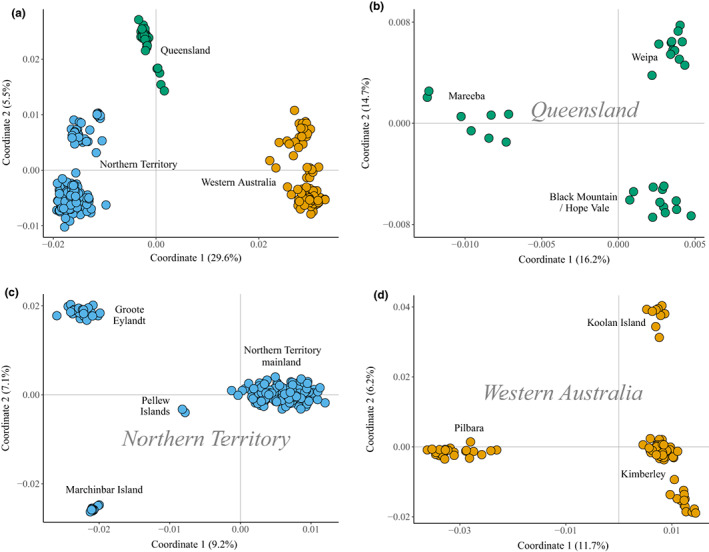
Principal coordinate plots of genomic distance between northern quoll individuals. (a) Genomic distances between all individuals, and (b–d) genomic distances within one of each of the three major clusters observed in (a). Panel (b) includes individuals from Queensland; (c) includes individuals from the Northern Territory; and (d) individuals from Western Australia. The percentage of the total variance explained by each coordinate is shown on axis labels

The principal coordinates plot identified three predominant clusters (with some substructure, Figure 2) corresponding with known major biogeographical barriers that separate the QLD and NT (Carpentarian Gap), and NT and WA (Ord Arid Region), regions of the species distribution. The first and second coordinate dimensions explained 33.3% and 11.8% of the total variance, respectively. In the NT, island populations all separated independently from the mainland populations (Figure 2b). Queensland populations showed a clear east–west separation, with the Weipa population on the western coast of Cape York separating strongly from the east coast populations (Figure 2c). In WA, there were two broad clusters corresponding to the Kimberley and the Pilbara regions (Figure 2d). Some substructuring in the Kimberley region of WA suggests isolation of Koolan Island and Mornington Sanctuary from the other populations.

**FIGURE 3:**
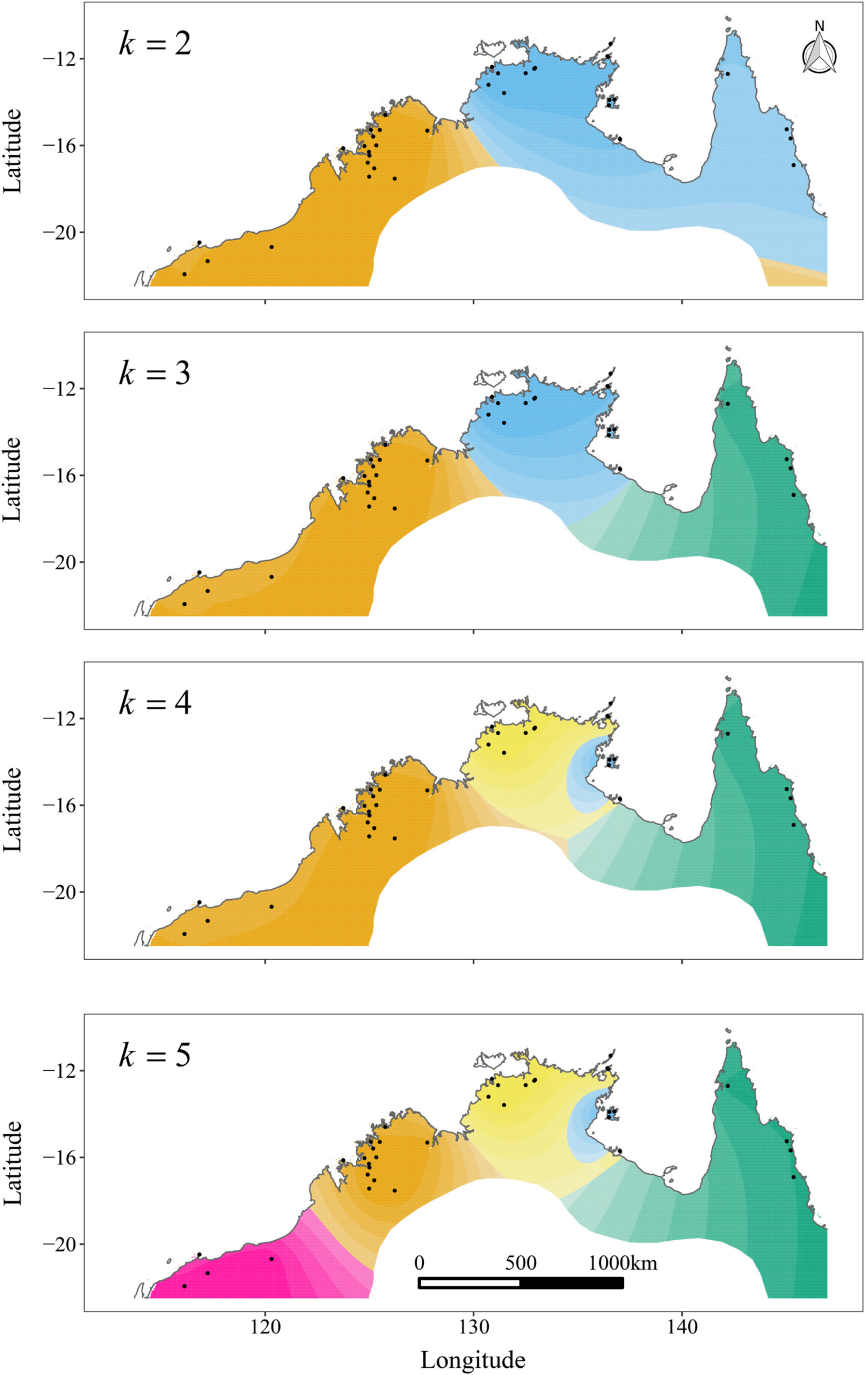
Patterns of landscape genomic structure across the geographical range of the northern quoll. Each panel shows the population genomic structure when two, three, four or five ancestral clusters (*k* values) are identified in the data set. Colours in each panel represent the distribution of an ancestral cluster, interpolated across the historical distribution of the species. Black points indicate sampling locations

There was a clear pattern of hierarchical population genomic structure in the data set, with increasing values of *k* populations resulting in decreasing values of the cross‐entropy criterion (Figure S4). The largest drop in cross‐entropy occurred between *k* = 1 and *k* = 2, followed by the drop between *k* = 2 and *k* = 3, suggesting that a value of 2 or 3 is most useful for describing the high‐level genetic structure across the species. At *k* = 2, the WA populations separated from the combined NT and QLD populations (Figure 3). At *k* = 3, the QLD and WA populations separated. Increasing the *k* value to 4 resulted in the Groote Eylandt populations separating from the rest of the NT, and increasing *k* to 5 resulted in the separation of the Pilbara and Kimberley regions of WA. When genomic structure was interpolated across the landscape, the boundaries between ancestral clusters strongly matched the locations of known biogeographical barriers (Figure 3). Higher values of *k* separated out other island groups, with *k* = 6 identifying Koolan Island in WA and *k* = 7 highlighting Marchinbar Island in the NT (Figure S5).

Values of genomic diversity metrics varied substantially across localities (Table 1). While low values of heterozygosity metrics were expected (because we used a low minor allele count threshold during filtering), some populations exhibited extremely low levels of diversity (e.g., Marchinbar Island; SNP *H*_E_ = 0.001), whereas others were considerably more diverse (e.g., Bachsten Creek; SNP *H*_E_ = 0.056) (Tables S5 and S6). Autosomal heterozygosity and SNP heterozygosity were highly correlated for both *H*_E_ and *H*_O_ (Pearson's product‐moment correlation; *r*_HE_ = 0.87, *r*_HO_ = 0.89; *p* < .001 for both parameters) (Figure S6).

**TABLE 1:** Population genomic parameters for the northern quoll (*Dasyurus hallucatus*)

Locality	Treatment	Region	*n*	*A*	*A*_E_	*H*_E_ (SNP)	*H*_O_ (SNP)	*H*_E_ (auto × 100)	*H*_O_ (auto × 100)	*F*_IS_	*P*_E_
Darwin	Naïve	NT	9	1.175	1.066	0.046	0.044	0.007	0.007	0.018	0.043
Outer Darwin	Naïve	NT	44	1.298	1.069	0.048	0.044	0.007	0.007	0.040	0.047
East Alligator (2003)	Naïve	NT	13	1.181	1.065	0.044	0.042	0.007	0.007	0.024	0.042
Kapalga	Naïve	NT	20	1.267	1.068	0.048	0.043	0.008	0.008	0.055	0.046
Artesian Range	Naïve	WA	19	1.310	1.081	0.057	0.056	0.012	0.012	0.017	0.055
Bachsten Creek	Naïve	WA	9	1.230	1.080	0.056	0.056	0.011	0.012	0.001	0.053
Wunaamin Miliwundi E	Naïve	WA	11	1.224	1.080	0.055	0.054	0.011	0.011	0.011	0.052
Wunaamin Miliwundi W	Naïve	WA	12	1.247	1.080	0.056	0.055	0.011	0.011	0.005	0.053
Millstream Chichester NP	Naïve	WA	8	1.137	1.066	0.044	0.044	0.008	0.008	−0.013	0.041
Mornington Sanctuary	Naïve	WA	15	1.169	1.073	0.047	0.046	0.009	0.009	0.020	0.045
Marble Bar	Naïve	WA	8	1.130	1.064	0.042	0.043	0.008	0.008	−0.023	0.039
Black Mountain	Exposed	QLD	6	1.061	1.028	0.020	0.019	0.006	0.006	0.005	0.018
East Alligator (2014)	Exposed	NT	6	1.140	1.066	0.045	0.041	0.006	0.006	0.057	0.041
East Alligator (2017)	Exposed	NT	8	1.129	1.061	0.041	0.040	0.006	0.006	0.013	0.038
Hope Vale	Exposed	QLD	6	1.065	1.030	0.021	0.020	0.006	0.006	0.003	0.019
Mareeba	Exposed	QLD	8	1.075	1.032	0.022	0.021	0.006	0.006	0.017	0.020
Weipa	Exposed	QLD	12	1.034	1.018	0.011	0.012	0.003	0.004	−0.023	0.011
Groote West	Island	NT	16	1.080	1.041	0.026	0.023	0.004	0.004	0.108	0.025
Koolan Island	Island	WA	9	1.080	1.047	0.029	0.029	0.005	0.005	0.011	0.028
Marchinbar Island	Island	NT	19	1.005	1.002	0.001	0.002	0.000	0.000	−0.059	0.001
Astell Island (2005)	Translocated	NT	16	1.185	1.065	0.044	0.043	0.006	0.007	0.010	0.042
Astell Island (2016)	Translocated	NT	13	1.182	1.066	0.045	0.042	0.007	0.007	0.041	0.043
Pobassoo Island	Translocated	NT	15	1.156	1.062	0.041	0.040	0.006	0.007	0.017	0.040

*Note*: Sampling localities have been categorized into treatments according to their history with the presence of cane toads. Parameters include the sample size (*n*), number of alleles (*A*), effective number of alleles (*A*_E_), SNP expected heterozygosity (*H*_E_), SNP observed heterozygosity (*H*_O_), autosomal expected heterozygosity (*H*_E_ auto), autosomal observed heterozygosity (*H*_O_ auto), Wright's inbreeding coefficient (*F*_IS_) and the locus polymorphic index (*P*_E_). Localities have been ordered by treatment rather than geographical location. Standard errors and standard deviations for all parameters can be found in Tables S2 and S3, respectively.

Patterns of pairwise genomic differentiation across the landscape broadly corresponded to the pattern of geographical distance between localities (Figure S7), with the plot of isolation‐by‐distance showing a strong relationship between genetic differentiation and geographical distance (*z* = 38,952, *p* < .001). Analysis of spatial autocorrelation of genotypes identified significant values of the autocorrelation coefficient *r* persisting for distances exceeding 500 km (Figure S8). Seawater barriers substantially increased the differentiation of island populations from those on the nearby mainland. In particular, Marchinbar Island showed a very high level of differentiation from all other localities (*F*_ST_ = 0.5–0.9), probably due to its very low level of diversity (Figure S9). All pairwise comparisons of *F*_ST_ had a significant (*p* < .001) level of differentiation, based on 1000 bootstraps across loci, even where the same locality was sampled only a few years apart (although in such cases the magnitude of differentiation was very low, e.g., East Alligator). The *F*_ST_ values were very similar to the $G_{\mathrm{ST}}^{\prime \prime }$ values (Figure S10), with the Mantel test showing that our two pairwise matrices of differentiation metrics were strongly correlated (*z* = 15.57, *p* < .001).

### 3.3 Demographic and temporal trends

**FIGURE 4:**
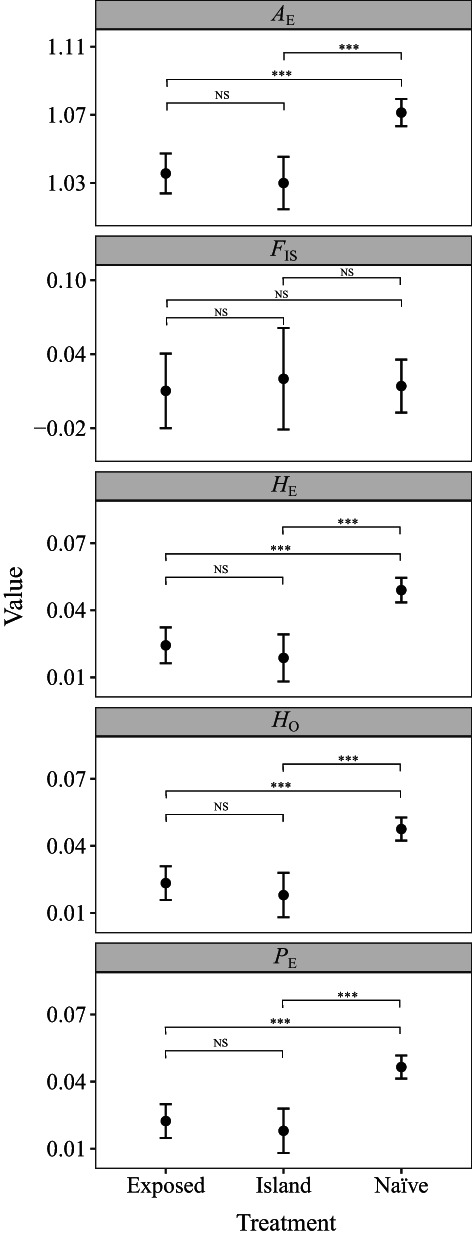
Effect of three treatments on five northern quoll population genomic parameters. Parameters include the effective number of alleles (*A*_E_), inbreeding coefficient (*F*_IS_), expected heterozygosity (*H*_E_), observed heterozygosity (*H*_O_) and the locus polymorphic index (*P*_E_). Treatments include populations that naturally occur on toad‐free islands (Island), occur on the mainland and have not been exposed to cane toads (Naïve), or occur on the mainland but have been exposed to cane toads (Exposed). Significance codes: ***, .001; **, .01; *, .05; NS, not significant

We found that three of the analysed population genomic parameters varied significantly among treatments (Figure 4). The generalized least square models showed that *H*_E_ (*χ* ^2^ = 40.5, *df* = 2, *p* < .001), *A*_E_ (*χ* ^2^ = 37.8, *df* = 2, *p* < .001) and *P*_E_ (*χ* ^2^ = 42.2, *df* = 2, *p* < .001) all differed significantly among the three (island, toad‐exposed and toad‐naïve) treatments. There was no significant difference in *F*_IS_ among treatments (*χ* ^2^ = 0.14, *df* = 2, *p* = .93). Tukey *post hoc* tests showed that toad‐naïve localities (mean *H*_E_ = 0.049) had significantly higher *H*_E_ than toad‐exposed (mean *H*_E_ = 0.024) and island (mean *H*_E_ = 0.019) localities (*t* = −5.0 to −5.1, *df* = 16.6 to 17.0, *p* < .001), with no significant difference between island and toad‐exposed localities (*t* = 0.83, *df* = 16.5, *p* = .22). Values of *H*_O_, *A*_E_ and *P*_E_ among treatments showed similar patterns to *H*_E_ (Table S7). For example, toad‐naïve localities (mean *A*_E_ = 1.072) had significantly higher *A*_E_ than toad‐exposed (mean *A*_E_ = 1.035) and island (mean *A*_E_ = 1.03) localities (*t* = −4.7 to −5.0, *df* = 16.6 to 17.0, *p* < .001), with no significant difference in *A*_E_ between island and toad‐exposed localities (*t* = 0.57, *df* = 16.6, *p* = .84).

Temporally replicated localities showed some significant changes through time, corresponding with isolation on islands or toad invasion and establishment (Table 1). For example, over the 11‐year interval between sampling events on Astell Island (where an insurance population of the northern quoll was established), the *F*_IS_ value for the SNP data set increased from 0.01 to 0.04 (*t* = 3.434, *p* < .001). This is despite only minor changes in *H*_E_ (*t* = 0.595, *p* = .552) and *H*_O_ (*t* = −0.637, *p* = .524) over the same period. At the East Alligator locality between 2003 and 2017, where cane toads invaded between sampling periods, there was a significant reduction in *P*_E_ (*t* = −3.01, *p* = .003) and *H*_E_ (*t* = −2.294, *p* = .02), but no change in *H*_O_ (*t* = −1.364, *p* = .173) or *F*_IS_ (*t* = −1.072, *p* = .284), based on the SNP data. Heterozygosity estimates calculated using the SNP data broadly agreed with heterozygosity estimates calculated from autosomal data (i.e., where all variant and invariant sites were analysed). However, the estimates calculated from SNP data showed slightly greater stochasticity than the autosomal estimates, probably due to sample size variation among populations/years. For example, SNP estimates of *H*_E_ at East Alligator increased slightly from 2003 to 2014, and then decreased from 2014 to 2017, whereas the autosomal estimates show a consistent reduction through time in *H*_E_ and *H*_O_, with both values declining from 7e^−5^ to 6e^−5^ between 2003 and 2014, and no change observed between 2014 and 2017. As the autosomal estimates are likely to be a more robust measure of heterozygosity, we suggest this pattern is more realistic.

**FIGURE 5:**
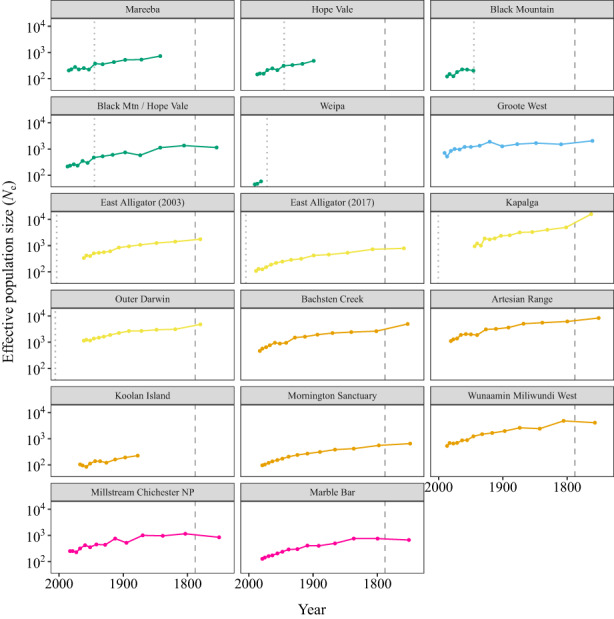
Recent effective population size (*N*_*e*_) trajectories inferred at 14 sampling localities of the northern quoll. Colours match those used in Figure 3 (*k* = 5). The vertical dashed grey line in each panel represents the start of European colonization of Australia in 1788, with the dotted grey line representing the arrival of the cane toad invasion front at a locality. Note the log scale used on the *y*‐axis

Investigation of historical demographics using *snep* suggested that there have been strong declines in the effective population sizes (*N*_*e*_) of many northern quoll populations for much of the last century (Figure 5). Localities showing the largest declines in *N*_*e*_ include the NT populations of outer Darwin and Kapalga, and the WA populations of Artesian Range and Bachsten Creek. Island populations (e.g., Groote Eylandt and Koolan Island) tended to show smaller but somewhat more consistent population sizes through time than mainland populations. The toad‐exposed QLD localities were characterized by small effective population sizes for much of the past century, with noticeable reductions in *N*_*e*_ after arrival of cane toads into Mareeba, Hope Vale and the combined Hope Vale/Black Mountain localities. However, there did not appear to be enough genomic diversity in the sequence data for the Weipa locality to provide *snep* with the information content required to see past the arrival of cane toads to the area.

**FIGURE 6:**
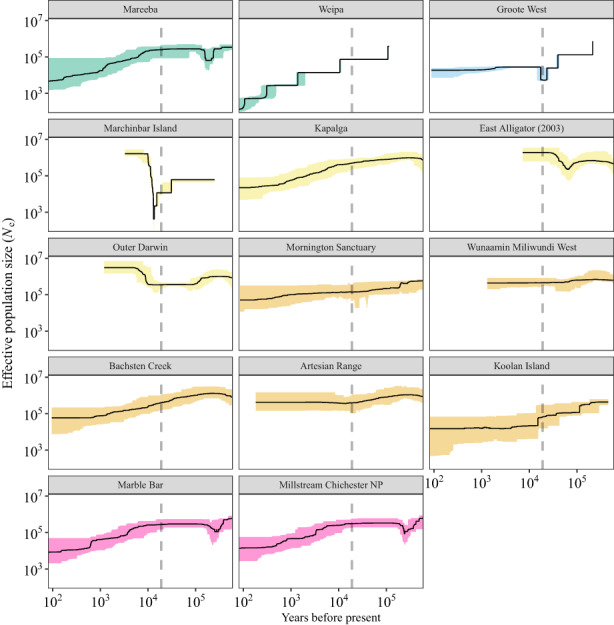
Stairway plots of estimated historical effective population size (*N*_*e*_) at 14 sampling localities of the northern quoll. The solid black line in each panel represents the median estimate of *N*_*e*_, with shaded regions representing 95% confidence intervals. Colours match those used in Figure 3 (*k* = 5). The vertical dashed grey line in each panel represents the end of the last glacial maximum about 19 000 years before present. Note the log scale used on both axes

Stairway plots showed expected fluctuations in *N*_*e*_ of island populations around the time of isolation due to sea‐level rise after the end of the last glacial maximum (Figure 6), suggesting that they were useful for interpretation of population sizes over much deeper time periods than *snep* . Broadly, the stairway plots showed similar trends to those produced by *snep* , with long, slow declines at many mainland localities and more stable populations on islands (after the last glacial maximum). The EBSPs showed considerably less certainty with estimated population sizes, highlighted by the large confidence intervals for all localities that were analysed and mediocre convergence (posterior effective sample size) in some localities (e.g., Groote West). Results suggested recent population declines in most populations, consistent with the outputs from *snep* and Stairway Plot 2 (Figure S11), with the posterior distributions of the number of population size changes indicating that the constant population size hypothesis could be rejected for all populations (Table S4). While we were unable to scale the temporal axis for the EBSPs, the WA populations showed weaker declines than suggested by *snep* , suggesting that, as for the stairway plots, the deeper‐time analysis using ESBPs may not be observing very recent declines (e.g., last 50–100 years). This was most pronounced for populations in the highly remote and rugged areas of the western Kimberley, where threats may have developed more recently.

## 4 DISCUSSION

While biodiversity declines are well recognized as a global problem, the consequences of these declines for patterns of genomic diversity are often poorly known. Using genomic data to infer recent and long‐term historical demographic trends can help identify the extent to which loss of genomic diversity itself is threatening species' persistence. Northern Australia's vast savannas are the location of some of the world's most dramatic recent declines of mammals (Cremona et al., 2022; Woinarski, Legge, et al., 2011), and here we illustrate patterns of genomic diversity and long‐term trajectories for a keystone declining species, the northern quoll. Two major influences appear to be driving the observed patterns of northern quoll population genomic structure and diversity across the Australian continent. First, major known biogeographical barriers are responsible for strong hierarchical population structure resulting from patterns of vicariance and long‐term isolation. Second, it seems that the effective population size of many populations has been declining over much of the past century, in some cases exacerbated by the rapid range expansion of the introduced and lethally toxic cane toad. While ongoing longitudinal analysis of northern quoll populations will help disentangle the impacts of historical biogeography from the impacts of cane toad invasion on population genomic diversity, it appears as though multidecadal exposure to the cane toad is associated with a considerable reduction in heterozygosity. This is approximately equivalent to the loss of diversity on islands that have been isolated from the mainland for >10,000 years. Together, these results warn that ongoing and future declines of mammal populations in northern Australia resulting from anthropogenic pressures and introduced species may have long‐term impacts on species‐wide genomic diversity, despite remnant populations persisting in some localities.

This data set represents the most comprehensive tissue sampling of a mammal across northern Australia to date and is thus an important quantification of the genomic impacts of regional mammal declines. Our data suggest that effective population sizes have been declining even in the absence of cane toads, suggesting that cane toads represent a compounding threat for northern quoll populations. While many areas are lacking quantitative historical data on population sizes, our results agree with a small number of studies and anecdotal data suggesting that northern quoll populations were indeed declining prior to the introduction and spread of cane toads (Braithwaite & Griffiths, 1994; Woinarski et al., 2008). Such declines have also been occurring in a range of other native mammal species across northern Australia (Fisher et al., 2014; Penton et al., 2021; von Takach et al., 2020; Woinarski et al., 2010). This massive conservation problem appears to be driven by a suite of interacting factors, including habitat degradation due to feral herbivores and pastoralism (Legge et al., 2011), increased predation due to feral cats (*Felis catus*) (Frank et al., 2014), frequent or high‐intensity fires (von Takach et al., 2020, 2022), and possibly climate change (Kutt et al., 2009; Traill et al., 2011).

These broad drivers of decline contribute to isolation of remnant populations and reduction of gene flow, both of which can lead to reductions in effective population size. Unfortunately, the introduction and spread of cane toads appears to have exacerbated these threats and is causing a major loss of diversity as cane toads spread across the Australian mainland. This loss of diversity probably results from the combination of strong selection on genotypes expressing avoidance behaviours (Kelly & Phillips, 2017) as well as the severe reductions in northern quoll population sizes when cane toads arrive in an area (Burnett, 1997; Woinarski et al., 2010).

In this context, future monitoring and tissue sampling of northern quoll populations in the Kimberley and Top End regions of northern Australia will be useful for quantifying how genomic diversity is impacted as population sizes decline with the expansion of cane toad populations. Cane toads are now well established across the Top End and have almost completely occupied the Kimberley region. While we did not observe significant increases in the inbreeding coefficient (*F*_IS_) in toad‐exposed populations, it is worth noting that this metric does not provide information on the cumulative level of inbreeding in a population, but rather detects nonrandom mating in the most recent generation (Waples, 2015). With further longitudinal sampling of impacted populations, investigation of alternative metrics (e.g., mean kinship coefficients within a population) may be warranted. Our Kimberley population genomic data provide a comprehensive baseline understanding of toad‐naïve populations in the region, and can be used in a before‐and‐after‐impact analysis framework. Ongoing sampling in the region will clarify the immediate impacts of cane toad invasion, but sampling over the next two to three decades will be vital for understanding the temporal pattern of the loss of genomic diversity.

The implication that mammal declines may have been occurring for long periods of time (prior to the start of widespread wildlife monitoring efforts) could help quantify the relative importance of recent (<50 years) changes in landscape management as drivers of decline. For example, while feral cats have probably been present for ~150 years (Abbott et al., 2014), the impacts of climate change have probably been occurring for much longer periods (Brüniche‐Olsen et al., 2014; White et al., 2018). Using genomic data to obtain information on historical effective population sizes is one valuable way to disentangle these drivers of demographic change. With genome‐wide sequencing data becoming cheaper and more readily accessible, we suggest that an important avenue of future work will be to conduct similar analyses on additional species with similar distributions, allowing us to compare and contrast patterns of decline between species.

As anthropogenic stressors impact ecological communities, declining species tend to show contractions in their realized niche space (Scheele et al., 2017). Populations within a subset of environmental conditions can thus exhibit stronger patterns of persistence (von Takach et al., 2022). For many Australian mammal species, the drivers of decline appear to be either mitigated or better tolerated on offshore islands (Legge et al., 2018). For example, while cane toads have shown uncontrolled spread across the mainland, they have not yet managed to establish viable populations on numerous islands. Some of these islands serve as useful conservation refuges for populations of northern quolls (How et al., 2009), including two islands onto which northern quolls have been translocated as a means of conservation management (Rankmore et al., 2008).

Our data show that islands certainly have a strong role to play in the conservation of genomic diversity, with WA and the NT home to remnant island populations of northern quolls that contain a portion of the species‐wide diversity. For many island lineages, this probably includes a small unique contribution of alleles to overall allelic richness (von Takach, Penton, et al., 2021). However, as is the case for many species and islands around the world, these populations tend to show reduced genetic diversity (Cardoso et al., 2009; Frankham, 1997; Spencer et al., 2017). For example, our Marchinbar Island samples had extremely low levels of genomic diversity, with just 59 of 10,191 SNPs being polymorphic among the 19 individuals. Hence, the usefulness of these remnant island populations for maintenance of genomic diversity and associated adaptive capacity is somewhat limited, and there may be a case for translocations between islands, and from the mainland, if these islands are to act as refugia for substantial portions of the available genetic diversity. Islands are, however, also not immune to invasion by cane toads. A sobering example is the extirpation of northern quolls on the Sir Edward Pellew Island group, after cane toads established populations by rafting on freshwater plumes and debris associated with a large flooding event on the McArthur River (Woinarski, Ward, et al., 2011).

While remnant island populations exhibit lower genetic diversity, our data also show that the process of translocating northern quolls to toad‐free islands as insurance populations has not led to a substantial reduction in genome‐wide diversity metrics. While changes in some metrics, such as effective population size, can take over 20 generations to be detectable in RADseq data (Nunziata & Weisrock, 2018), islands such as Astell Island have carrying capacities in the thousands of individuals (Griffiths et al., 2017), making drift a weaker force and allowing for the retention of high levels of genomic diversity. Such insurance populations require ongoing management and genetic monitoring, however, and even short‐term isolation of island populations from predators can result in the rapid loss of critical behavioural traits (Jolly et al., 2018; Jolly & Phillips, 2021). This may prevent successful reintroduction of island individuals back to the mainland into their former realized niche. Further, successful establishment into historically occupied niche space may be made more difficult due to the ecological release of interspecific competitors or the invasion of exotic species into vacated niche space (Doherty & Driscoll, 2018), factors that require consideration for future management actions.

Broadly, our data concur with previously observed phylogeographical studies, showing patterns of isolation resulting from biogeographical barriers such as the Great Sandy Desert, the Bonaparte Gulf/Ord Arid Region and the Carpentarian Gap. These barriers have variously been observed to limit dispersal in plants (Edwards et al., 2017), frogs (Catullo et al., 2014), reptiles (Melville et al., 2011), birds (Lee & Edwards, 2008) and mammals (Eldridge et al., 2014; Potter et al., 2012). Early collections of northern quolls, led Thomas (1926) to recognize four distinct forms, including *Dasyurus hallucatus hallucatus*, *D. h. nesaeus*, *D. h. exilis* and *D. h. predator*, with type localities from the Top End (NT), Groote Eylandt (NT), eastern Kimberley (WA) and Cape York (QLD), respectively (Mahoney & Ride, 1988). These subspecies have not been recognized by subsequent authors due to the apparent lack of distinct morphological differentiation (Jackson & Groves, 2015; Oakwood, 2008; Viacava et al., 2020), despite molecular evidence of two to four divergent lineages (Firestone, 2000; Woolley et al., 2015). Here, we present data showing strong hierarchical population structuring of these divergent lineages, which conforms to the disjunct nature of the northern quoll distribution across the continent. Ongoing morphological assessment will help to clarify differences in phenotype between these lineages (Umbrello, 2018). Another direction currently being investigated is to use exon capture data to investigate in greater detail the level and timing of divergence between the major lineages presented here (Eldridge et al., 2020). Further investigation of the functional and adaptive significance of putatively adaptive loci will complement such findings, with future genome annotation probably able to provide compelling evidence for agents of selection, physiological processes, phenotypes and the occurrence of selective sweeps (Pardo‐Diaz et al., 2015).

Together, our data suggest that several critical aspects need consideration for the management of northern quoll populations across the Australian continent. These include (i) that biogeographical barriers separate subregions each containing substantial amounts of unique genomic diversity; (ii) that remnant island populations harbour only a subset of the diversity present within each subregion; (iii) that large insurance populations on islands or in intensively managed/fenced safe havens can maintain high levels of genomic diversity (although these populations may lose adaptive traits and require ongoing genetic management) and (iv) that declines induced by cane toads are dramatic, but play out against broader declines resulting from other anthropogenic stressors and threats. With the exception of cane toad impacts, these considerations are likely to be similar for many of the widespread faunal species within the Australian monsoonal tropics (Bowman et al., 2010). The dramatic impact of toads on population sizes of northern quolls points to the potential value of using offshore islands as insurance populations, and also to the value of preventing cane toads from colonizing the Pilbara region of Western Australia (Southwell et al., 2017). Our work also raises questions about whether there may be value in mixing populations across islands or between mainland and islands to secure the genetic diversity of quolls. Future work on quolls and other taxa will help to clarify the extent to which intraspecies conservation management needs to operate within and among lineages or evolutionarily significant units across northern Australia.

## AUTHOR CONTRIBUTIONS

SB, BvT and BLP designed the research with input from KO and DF. CPB, SFC, RH, EK, BLP, IJR, PBSS and GJT contributed to data collection and/or sample preparation. BvT and LR carried out the data analysis. BvT and SB interpreted the results with input from all authors. BvT wrote the paper with input, advice and contributions from all authors with respect to manuscript structure, framing and intellectual content.

## CONFLICT OF INTEREST

The authors declare no conflict of interest.

## BENEFIT‐SHARING STATEMENT

A research collaboration was developed with researchers around Australia, who provided tissue samples for genetic analysis. All collaborators were offered the opportunity to contribute to the manuscript as co‐authors, and the results of research are available for the broader scientific community through online databases and open access publishing. The research addresses a priority concern, in this case the conservation genetics and management of the threatened northern quoll, and findings will be incorporated into management actions for the species. Bioplatforms Australia is a nonprofit organization that supports Australian Life science research by investing in state‐of‐the‐art infrastructure and expertise in genomics, proteomics, metabolomics and bioinformatics. Investment funding for Bioplatforms Australia is provided by the Commonwealth Government National Collaborative Research Infrastructure Strategy. The Bioplatforms Australia Oz Mammal Genomics Initiative is one of many national collaborative projects that generate high‐impact data and knowledge resources to support some of Australia's biggest scientific challenges (https://data.bioplatforms.com/about). These challenges span agriculture, biomedicine, the environment and industry, as well as extending to relevant international endeavours.

## Supporting information


Appendix S1

## Data Availability

All sequencing data have been uploaded to the Bioplatforms Australia Oz Mammal Genomics Initiative data portal (https://data.bioplatforms.com/organization/about/bpa‐omg) (data set IDs 102.100.100/52650 and 102.100.100/52623). These data are made available openly under a Creative Commons Attribution licence. All other data, scripts and sample metadata have been uploaded to the Dryad Digital Repository (DOI: 10.5061/dryad.wh70rxwr6). This includes (i) bioinformatic scripts and the draft *Dasyurus hallucatus* reference genome; (ii) outputs from angsd (including unfiltered genotypes); (iii) genotype data for historical demographic inference and (iv) R scripts for SNP filtering, genomic analyses and figure construction.
