# Supplementary material for: Population genomics of a predatory mammal reveals patterns of decline and impacts of exposure to toxic toads
Source: Mol Ecol. 2022 Sep 25;31(21):5468–86. doi: 10.1111/mec.16680 (PMC9826391; doi:10.1111/mec.16680)
Supplement: Supplementary file 1 — Appendix S1 [file MEC-31-5468-s001.docx]

**Supplemental Information for:**

**Population genomics of a predatory mammal reveals patterns of decline and impacts of exposure to toxic toads**

Brenton von Takach et al.

**Table of Contents:**

| **Table S1** | Pages 1-2 |
| --- | --- |
| **Table S2** | Pages 4-5 |
| **Table S3** | Page 6 |
| **Table S4** | Pages 7-10 |
| **Table S5** | Page 11 |
| **Table S6** | Page 12 |
| **Table S7** | Page 13 |
| **Figure S1** | Page 14 |
| **Figure S2** | Page 15 |
| **Figure S3** | Page 16 |
| **Figure S4** | Page 17 |
| **Figure S5** | Page 18 |
| **Figure S6** | Page 19 |
| **Figure S7** | Page 20 |
| **Figure S8** | Page 21 |
| **Figure S9** | Page 22 |
| **Figure S10** | Page 23 |
| **Figure S11** | Page 24 |

Table S1. List of all northern quoll sampling localities, where tissue was collected for next-generation sequencing and analysis. Localities listed twice or thrice had tissues collected in more than one sampling period and were separated for analyses. NT = Northern Territory, QLD = Queensland, and WA = Western Australia.

| Region | Population | Sample size | Year | Latitude | Longitude | Material | Treatment |
| --- | --- | --- | --- | --- | --- | --- | --- |
| NT | Alligator Floodplains | 2 | 2004 | -12.27 | 132.67 | liver | toad-exposed |
| NT | Astell Island | 17 | 2005 | -11.88 | 136.42 | ear | translocated |
| NT | Astell Island | 14 | 2016 | -11.89 | 136.43 | ear | translocated |
| NT | Darwin | 10 | 2000/2001 | -12.38 | 130.87 | NA | toad-naïve |
| NT | Darwin | 2 | 2019 | -12.38 | 130.87 | ear | toad-exposed |
| NT | East Alligator | 15 | 2003 | -12.41 | 132.96 | ear | toad-naïve |
| NT | East Alligator | 15 | 2014 | -12.43 | 132.94 | ear | toad-exposed |
| NT | East Alligator | 8 | 2017 | -12.43 | 132.95 | ear | toad-exposed |
| NT | Groote East | 12 | 2014 | -13.89 | 136.75 | blood | island |
| NT | Groote North | 6 | 2006 | -13.69 | 136.74 | ear | island |
| NT | Groote South | 9 | 2014 | -14.13 | 136.49 | blood | island |
| NT | Groote West | 16 | 2018 | -13.90 | 136.52 | ear | island |
| NT | Hayes Creek | 5 | 2003 | -13.58 | 131.46 | NA | toad-naïve |
| NT | Kapalga | 25 | 1994 | -12.67 | 132.52 | NA | toad-naïve |
| NT | Kapalga | 10 | 2014 | -12.65 | 132.37 | blood | toad-exposed |
| NT | Litchfield National Park | 4 | 2003 | -13.20 | 130.71 | NA | toad-naïve |
| NT | Marchinbar Island | 28 | 2004 | -11.30 | 136.59 | ear | island |
| NT | Oenpelli Road | 11 | 2004 | -12.47 | 132.87 | NA | toad-naïve |
| NT | Outer Darwin | 53 | 2003 | -12.66 | 131.17 | NA | toad-naïve |
| NT | Pellew Islands | 2 | NA | -15.71 | 137.03 | liver | island |
| NT | Pobassoo Island | 16 | 2005 | -11.92 | 136.45 | NA | translocated |
| NT | South Alligator | 4 | 2013 | -12.69 | 132.47 | ear | toad-exposed |
| QLD | Black Mountain | 6 | 2015/2016 | -15.68 | 145.21 | ear | toad-exposed |
| QLD | Hope Vale | 6 | 2016 | -15.25 | 145.02 | ear | toad-exposed |
| QLD | Mareeba | 8 | 2015 | -16.90 | 145.36 | ear | toad-exposed |
| QLD | Townsville | 1 | 2001 | -19.58 | 147.03 | ear | toad-exposed |
| QLD | Weipa | 12 | 2018 | -12.70 | 142.20 | ear | toad-exposed |
| WA | Artesian Range | 20 | 2013 | -16.46 | 125.01 | ear | toad-naïve |
| WA | Bachsten Creek | 9 | 2013 | -15.99 | 125.33 | ear | toad-naïve |
| WA | Dolphin Island | 12 | 2012/2013 | -20.47 | 116.85 | ear | island |
| WA | King Cascade | 1 | 2009 | -15.59 | 125.18 | ear | toad-naïve |
| WA | Wunaamin Miliwundi East | 11 | 2014/2015 | -17.06 | 125.23 | ear | toad-naïve |
| WA | Wunaamin Miliwundi West | 14 | 2016 | -16.79 | 124.92 | ear | toad-naïve |
| WA | Koolan Island | 10 | 2006/2007 | -16.13 | 123.75 | ear | island |
| WA | Millstream Chichester NP | 11 | 2014 | -21.33 | 117.24 | ear | toad-naïve |
| WA | Mitchell Plateau | 3 | 2012 | -14.78 | 125.76 | ear | toad-naïve |
| WA | Mornington Sanctuary | 18 | 2012 | -17.53 | 126.22 | ear | toad-naïve |
| WA | Mount French | 2 | 2009 | -16.03 | 124.76 | ear | toad-naïve |
| WA | Northwest Marble Bar | 9 | 2011/2012 | -20.68 | 120.31 | ear | toad-naïve |
| WA | Oombulgurri | 1 | NA | -15.32 | 127.78 | ear | toad-naïve |
| WA | Pannawonica | 9 | 2012/2013 | -21.90 | 116.13 | ear | toad-naïve |
| WA | Prince Regent | 1 | 2012 | -15.28 | 125.07 | ear | toad-naïve |
| WA | Roe River Mouth | 5 | 2009 | -15.28 | 125.50 | ear | toad-naïve |
| WA | UCL | 2 | 2012 | -22.03 | 116.54 | ear | toad-naïve |
| WA | Walcott Inlet | 2 | 2014 | -16.30 | 124.98 | ear | toad-naïve |
| WA | Windjana Gorge | 2 | 2014 | -17.44 | 124.99 | ear | toad-naïve |

Table S2. The genomic location (chromosome/scaffold ID number and starting position) of all RAD loci used to infer demographic history via extended Bayesian skyline plots, for six sampled localities of northern quolls. Note that these positions are based on the draft genome assembly.

| Population | Scaffold | Position |
| --- | --- | --- |
| Bachsten Creek | 36 | 13395686 |
| Bachsten Creek | 69 | 12584606 |
| Bachsten Creek | 69 | 98189480 |
| Bachsten Creek | 70 | 227659132 |
| Bachsten Creek | 71 | 4213126 |
| Bachsten Creek | 130 | 24382059 |
| Bachsten Creek | 136 | 19562531 |
| Bachsten Creek | 147 | 7520464 |
| Bachsten Creek | 266 | 19482659 |
| Bachsten Creek | 397 | 4225 |
| East Alligator (2003) | 53 | 32412090 |
| East Alligator (2003) | 53 | 172210652 |
| East Alligator (2003) | 67 | 64273118 |
| East Alligator (2003) | 70 | 224886787 |
| East Alligator (2003) | 100 | 39129186 |
| East Alligator (2003) | 101 | 379475 |
| East Alligator (2003) | 138 | 6572399 |
| East Alligator (2003) | 141 | 4690916 |
| East Alligator (2003) | 185 | 16420243 |
| East Alligator (2003) | 266 | 3022681 |
| Groote West | 67 | 5493803 |
| Groote West | 91 | 84977608 |
| Groote West | 100 | 35464996 |
| Groote West | 101 | 20283095 |
| Groote West | 101 | 31408466 |
| Groote West | 128 | 10447200 |
| Groote West | 132 | 31837064 |
| Groote West | 139 | 3819242 |
| Groote West | 171 | 3227 |
| Groote West | 222 | 67156921 |
| Mareeba | 36 | 200921004 |
| Mareeba | 100 | 7236032 |
| Mareeba | 101 | 1767422 |
| Mareeba | 101 | 14280378 |
| Mareeba | 101 | 27328942 |
| Mareeba | 129 | 27462088 |
| Mareeba | 130 | 25686725 |
| Mareeba | 140 | 1109491 |
| Mareeba | 197 | 1277 |
| Mareeba | 397 | 4225 |
| Millstream Chichester NP | 36 | 25218959 |
| Millstream Chichester NP | 53 | 32412090 |
| Millstream Chichester NP | 67 | 2017866 |
| Millstream Chichester NP | 71 | 10214666 |
| Millstream Chichester NP | 100 | 1346698 |
| Millstream Chichester NP | 100 | 40291589 |
| Millstream Chichester NP | 101 | 6603904 |
| Millstream Chichester NP | 142 | 5094189 |
| Millstream Chichester NP | 171 | 3227 |
| Millstream Chichester NP | 259 | 40011844 |
| Weipa | 67 | 5493803 |
| Weipa | 69 | 98189480 |
| Weipa | 91 | 7407449 |
| Weipa | 91 | 23422993 |
| Weipa | 94 | 4276385 |
| Weipa | 130 | 13613733 |
| Weipa | 139 | 3237690 |
| Weipa | 185 | 11945876 |
| Weipa | 397 | 4225 |

Table S3. Summary of data and parameters used to infer demographic history via extended Bayesian skyline plots. The effective sample size (ESS) is a measure of the quality of the estimate of the posterior distribution, with higher values indicating better estimates. The sum(indicators.alltrees) parameter indicates the posterior distributions of the number of population size changes.

| **Population** | **Number of RAD loci** | **Number Of SNPs per RAD locus** | **MCMC length** | **Convergence (Posterior ESS)** | **Sum(indicators.alltrees) posterior mean and [95% HPD]** |
| --- | --- | --- | --- | --- | --- |
| Bachsten Creek | 10 | 2-7 | 15,000,000,000 | >200 | 1.483 [1, 3] |
| East Alligator (2003) | 10 | 2-6 | 15,000,000,000 | >600 | 2.251 [1, 4] |
| Groote West | 10 | 2-4 | 40,000,000,000 | >100 | 2.946 [2, 4] |
| Mareeba | 10 | 2-4 | 15,000,000,000 | >400 | 2.884 [2, 5] |
| Millstream Chichester NP | 10 | 2-7 | 15,000,000,000 | >200 | 3.639 [2, 5] |
| Weipa | 9 | 2-5 | 15,000,000,000 | >200 | 2.963 [2, 4] |

Table S4. Locations (genomic scaffold ID and base pair position) of all candidate SNPs under putative selection in the northern quoll. Candidate SNPs were identified using either outlier analysis or latent factor mixed model analysis, where a “Yes” in either column indicates that the SNP was identified using the method and a “No” indicates it was not identified using the method. A two-column page layout has been used for ease of readability. Note that these positions are based on the draft *Dasyurus hallucatus* genome assembly.

| Scaffold | Position | LFMM | Outlier |
| --- | --- | --- | --- |
| 36 | 10947231 | Yes | Yes |
| 36 | 13210351 | Yes | No |
| 36 | 14958191 | Yes | Yes |
| 36 | 22617023 | Yes | Yes |
| 36 | 29712060 | Yes | No |
| 36 | 30477679 | Yes | No |
| 36 | 54699367 | Yes | No |
| 36 | 54903620 | No | Yes |
| 36 | 78658866 | Yes | Yes |
| 36 | 78758277 | Yes | Yes |
| 36 | 78758367 | Yes | Yes |
| 36 | 84259204 | Yes | No |
| 36 | 97067243 | Yes | No |
| 36 | 108839875 | Yes | No |
| 36 | 122063265 | Yes | Yes |
| 36 | 123635807 | Yes | No |
| 36 | 126617141 | Yes | Yes |
| 36 | 129964763 | Yes | Yes |
| 36 | 135181685 | No | Yes |
| 36 | 152368635 | Yes | Yes |
| 36 | 158491969 | Yes | No |
| 36 | 177456492 | No | Yes |
| 36 | 180692439 | Yes | No |
| 36 | 185883593 | Yes | No |
| 36 | 192034250 | No | Yes |
| 36 | 199996947 | No | Yes |
| 36 | 200737141 | No | Yes |
| 36 | 205916921 | Yes | No |
| 36 | 206516582 | Yes | No |
| 36 | 208564974 | No | Yes |
| 36 | 209494296 | Yes | Yes |
| 36 | 231612852 | Yes | No |
| 36 | 235546136 | Yes | No |
| 36 | 235674410 | Yes | No |
| 36 | 235674435 | No | Yes |
| 36 | 238574671 | Yes | No |
| 36 | 262031834 | Yes | No |
| 36 | 264794695 | Yes | Yes |
| 36 | 273494556 | Yes | Yes |
| 36 | 279620850 | Yes | No |
| 36 | 286145740 | Yes | No |
| 36 | 289534840 | Yes | No |
| 36 | 291175231 | No | Yes |
| 36 | 297135202 | Yes | Yes |
| 36 | 297786350 | No | Yes |
| 53 | 6941041 | Yes | Yes |
| 53 | 7482073 | Yes | Yes |
| 53 | 10577172 | Yes | No |
| 53 | 13001516 | Yes | No |
| 53 | 19044410 | Yes | Yes |
| 53 | 28936022 | Yes | Yes |
| 53 | 35320043 | No | Yes |
| 53 | 42585555 | Yes | Yes |
| 53 | 46660203 | No | Yes |
| 53 | 49878155 | No | Yes |
| 53 | 57520257 | No | Yes |
| 53 | 64693931 | Yes | Yes |
| 53 | 67079126 | No | Yes |
| 53 | 68552381 | Yes | Yes |
| 53 | 75124042 | Yes | No |
| 53 | 76258573 | Yes | Yes |
| 53 | 96738117 | Yes | Yes |
| 53 | 101990345 | Yes | No |
| 53 | 104569169 | Yes | Yes |
| 53 | 109210157 | Yes | Yes |
| 53 | 136740656 | Yes | No |
| 53 | 138869265 | Yes | Yes |
| 53 | 144615808 | Yes | No |
| 53 | 149470098 | No | Yes |
| 53 | 176577369 | Yes | No |
| 53 | 178358223 | Yes | Yes |
| 53 | 182631548 | No | Yes |
| 53 | 185146261 | Yes | Yes |
| 53 | 189321891 | Yes | Yes |
| 53 | 195857141 | Yes | No |
| 53 | 199819584 | Yes | No |
| 53 | 201380629 | Yes | No |
| 53 | 202924153 | No | Yes |
| 67 | 756493 | Yes | No |
| 67 | 764618 | No | Yes |
| 67 | 2017942 | Yes | No |
| 67 | 2802972 | Yes | No |
| 67 | 5931937 | Yes | No |
| 67 | 13840159 | No | Yes |
| 67 | 22083244 | Yes | Yes |
| 67 | 26596961 | Yes | Yes |
| 67 | 36082197 | No | Yes |
| 67 | 79084914 | Yes | No |
| 67 | 83277954 | Yes | No |
| 67 | 89470135 | No | Yes |
| 67 | 91154866 | Yes | No |
| 67 | 92000705 | Yes | Yes |
| 67 | 92000711 | Yes | Yes |
| 67 | 100100614 | No | Yes |
| 67 | 100410989 | Yes | No |
| 67 | 105826836 | Yes | Yes |
| 67 | 117222005 | Yes | No |
| 67 | 125801644 | Yes | No |
| 67 | 128068729 | Yes | No |
| 67 | 131569471 | Yes | No |
| 68 | 11100155 | Yes | Yes |
| 68 | 11191680 | No | Yes |
| 68 | 22553459 | Yes | No |
| 68 | 32483665 | No | Yes |
| 68 | 34068133 | Yes | Yes |
| 68 | 38242657 | Yes | No |
| 68 | 53178430 | Yes | No |
| 68 | 54541969 | Yes | No |
| 68 | 58253072 | Yes | No |
| 68 | 58253074 | No | Yes |
| 68 | 68206822 | Yes | No |
| 68 | 90219388 | Yes | No |
| 68 | 92233050 | No | Yes |
| 68 | 98429442 | Yes | Yes |
| 68 | 106197849 | Yes | Yes |
| 68 | 116344165 | No | Yes |
| 69 | 1143237 | Yes | No |
| 69 | 2864911 | Yes | Yes |
| 69 | 5655513 | Yes | No |
| 69 | 5655523 | No | Yes |
| 69 | 10622350 | Yes | No |
| 69 | 11588322 | No | Yes |
| 69 | 11588407 | Yes | No |
| 69 | 15439843 | Yes | No |
| 69 | 25646112 | Yes | No |
| 69 | 33659497 | Yes | No |
| 69 | 39665528 | Yes | Yes |
| 69 | 46267565 | Yes | No |
| 69 | 47518017 | Yes | No |
| 69 | 47676990 | Yes | No |
| 69 | 51895151 | Yes | No |
| 69 | 51895245 | No | Yes |
| 69 | 55051436 | Yes | No |
| 69 | 55051527 | Yes | No |
| 69 | 60886785 | Yes | No |
| 69 | 75197189 | Yes | Yes |
| 69 | 88482879 | Yes | No |
| 69 | 91261553 | Yes | No |
| 69 | 92097442 | Yes | Yes |
| 69 | 92814993 | Yes | No |
| 69 | 95170841 | No | Yes |
| 69 | 97502170 | Yes | Yes |
| 69 | 97749661 | Yes | No |
| 69 | 98189501 | Yes | No |
| 69 | 103498658 | Yes | No |
| 70 | 1633083 | Yes | Yes |
| 70 | 4866359 | Yes | No |
| 70 | 7243997 | Yes | Yes |
| 70 | 7583066 | No | Yes |
| 70 | 9443606 | Yes | No |
| 70 | 19859258 | No | Yes |
| 70 | 33628334 | Yes | No |
| 70 | 33628345 | No | Yes |
| 70 | 39718426 | Yes | No |
| 70 | 42521753 | Yes | Yes |
| 70 | 48578710 | No | Yes |
| 70 | 49499346 | No | Yes |
| 70 | 56293859 | Yes | Yes |
| 70 | 60214132 | Yes | No |
| 70 | 70988472 | No | Yes |
| 70 | 97611603 | No | Yes |
| 70 | 101310032 | Yes | No |
| 70 | 104731632 | Yes | No |
| 70 | 107400002 | No | Yes |
| 70 | 108001496 | Yes | No |
| 70 | 114209227 | Yes | No |
| 70 | 116322887 | Yes | Yes |
| 70 | 123067513 | No | Yes |
| 70 | 131393927 | Yes | No |
| 70 | 149212325 | Yes | No |
| 70 | 150163215 | Yes | Yes |
| 70 | 151413745 | Yes | Yes |
| 70 | 161100589 | No | Yes |
| 70 | 163151745 | No | Yes |
| 70 | 172176764 | Yes | No |
| 70 | 177559355 | No | Yes |
| 70 | 180616134 | Yes | No |
| 70 | 181432755 | Yes | No |
| 70 | 193565015 | No | Yes |
| 70 | 206766053 | Yes | No |
| 70 | 210179706 | Yes | No |
| 70 | 214189436 | Yes | No |
| 70 | 214922168 | No | Yes |
| 70 | 218518042 | Yes | No |
| 70 | 222335048 | Yes | Yes |
| 70 | 227659146 | Yes | No |
| 70 | 227760303 | Yes | No |
| 70 | 229143503 | Yes | Yes |
| 71 | 1044270 | Yes | No |
| 71 | 3895657 | Yes | No |
| 71 | 3895763 | No | Yes |
| 71 | 7127019 | Yes | No |
| 71 | 9074394 | Yes | No |
| 71 | 20985062 | No | Yes |
| 71 | 23846241 | Yes | No |
| 71 | 27877503 | Yes | No |
| 71 | 28097861 | Yes | No |
| 71 | 35312881 | Yes | No |
| 71 | 47822383 | No | Yes |
| 71 | 50707403 | Yes | Yes |
| 71 | 57081695 | Yes | Yes |
| 71 | 60876942 | No | Yes |
| 71 | 62858729 | Yes | No |
| 71 | 81794609 | No | Yes |
| 72 | 12690277 | No | Yes |
| 72 | 12989864 | No | Yes |
| 72 | 29251285 | No | Yes |
| 72 | 29381510 | Yes | No |
| 72 | 30129349 | Yes | No |
| 72 | 35407343 | Yes | Yes |
| 72 | 42454340 | Yes | Yes |
| 72 | 42454407 | No | Yes |
| 72 | 48849872 | Yes | No |
| 72 | 51353675 | Yes | No |
| 72 | 53334813 | Yes | Yes |
| 72 | 57358466 | No | Yes |
| 72 | 65477671 | No | Yes |
| 72 | 75742300 | Yes | No |
| 72 | 110217283 | Yes | Yes |
| 72 | 110217325 | No | Yes |
| 72 | 114811669 | No | Yes |
| 72 | 116811222 | Yes | No |
| 72 | 127744078 | Yes | No |
| 75 | 1528115 | Yes | No |
| 75 | 7393416 | No | Yes |
| 75 | 16572995 | Yes | Yes |
| 75 | 17050559 | No | Yes |
| 75 | 18672175 | Yes | No |
| 75 | 21789326 | No | Yes |
| 75 | 27509435 | Yes | No |
| 75 | 36969724 | Yes | No |
| 75 | 38534521 | No | Yes |
| 75 | 69246519 | No | Yes |
| 75 | 74194283 | Yes | No |
| 75 | 78362014 | Yes | Yes |
| 75 | 79412995 | Yes | No |
| 75 | 88096422 | Yes | Yes |
| 76 | 3392887 | Yes | Yes |
| 76 | 3619049 | Yes | No |
| 76 | 20603166 | Yes | Yes |
| 76 | 23048482 | Yes | No |
| 76 | 25130067 | Yes | No |
| 89 | 7005875 | No | Yes |
| 90 | 17859234 | Yes | Yes |
| 90 | 17859263 | Yes | No |
| 90 | 29159192 | Yes | No |
| 91 | 2456435 | Yes | No |
| 91 | 5618740 | Yes | No |
| 91 | 7131361 | Yes | Yes |
| 91 | 7407485 | Yes | No |
| 91 | 9418119 | Yes | Yes |
| 91 | 9727658 | Yes | Yes |
| 91 | 15635632 | No | Yes |
| 91 | 23611732 | Yes | No |
| 91 | 33946196 | No | Yes |
| 91 | 46465794 | Yes | No |
| 91 | 53010149 | Yes | No |
| 91 | 63453516 | Yes | Yes |
| 91 | 68662310 | Yes | No |
| 91 | 80575555 | Yes | Yes |
| 91 | 82968734 | Yes | No |
| 91 | 83841242 | No | Yes |
| 91 | 91209196 | Yes | No |
| 94 | 4276420 | Yes | No |
| 94 | 7600837 | Yes | No |
| 94 | 21736469 | Yes | No |
| 94 | 24665479 | Yes | No |
| 94 | 34985515 | Yes | No |
| 94 | 36086736 | Yes | Yes |
| 94 | 50694530 | No | Yes |
| 94 | 52856162 | Yes | No |
| 94 | 55157252 | Yes | Yes |
| 94 | 62495237 | No | Yes |
| 96 | 1091938 | No | Yes |
| 96 | 4414628 | No | Yes |
| 96 | 11015123 | Yes | No |
| 96 | 13208522 | No | Yes |
| 96 | 27194634 | No | Yes |
| 96 | 28424168 | Yes | Yes |
| 96 | 39918862 | Yes | Yes |
| 96 | 41165169 | Yes | No |
| 96 | 74008234 | Yes | No |
| 96 | 74508170 | No | Yes |
| 96 | 77724533 | No | Yes |
| 96 | 82786227 | Yes | No |
| 97 | 1224147 | Yes | No |
| 97 | 7426563 | Yes | Yes |
| 98 | 1482518 | Yes | Yes |
| 98 | 16015379 | Yes | No |
| 98 | 16015426 | No | Yes |
| 98 | 16686968 | Yes | Yes |
| 98 | 27644973 | Yes | No |
| 98 | 35592241 | Yes | No |
| 98 | 37351323 | No | Yes |
| 98 | 40167304 | No | Yes |
| 98 | 59077737 | No | Yes |
| 98 | 60644091 | Yes | Yes |
| 98 | 66003524 | Yes | No |
| 98 | 74203989 | No | Yes |
| 99 | 7975617 | Yes | Yes |
| 99 | 9028633 | No | Yes |
| 100 | 1346817 | Yes | No |
| 100 | 4684637 | Yes | No |
| 100 | 7236054 | Yes | No |
| 100 | 13076274 | Yes | No |
| 100 | 15368094 | Yes | No |
| 100 | 18188283 | Yes | No |
| 100 | 19101578 | No | Yes |
| 100 | 19771999 | Yes | No |
| 100 | 24593891 | Yes | Yes |
| 100 | 30403143 | Yes | No |
| 100 | 33522423 | No | Yes |
| 100 | 38609480 | Yes | No |
| 100 | 39129224 | Yes | No |
| 100 | 40291653 | Yes | No |
| 100 | 41489729 | No | Yes |
| 101 | 1767539 | Yes | No |
| 101 | 5480465 | No | Yes |
| 101 | 14817765 | Yes | Yes |
| 101 | 15464466 | Yes | No |
| 101 | 16161727 | Yes | No |
| 101 | 17338338 | Yes | Yes |
| 101 | 25933240 | Yes | No |
| 101 | 27116208 | No | Yes |
| 101 | 27328950 | Yes | No |
| 101 | 31408540 | Yes | Yes |
| 101 | 35661454 | Yes | Yes |
| 101 | 36226533 | Yes | Yes |
| 101 | 38996640 | Yes | No |
| 101 | 49471978 | No | Yes |
| 101 | 52569819 | Yes | No |
| 101 | 53075098 | Yes | Yes |
| 124 | 2999515 | Yes | No |
| 124 | 5047883 | Yes | Yes |
| 125 | 4433110 | Yes | No |
| 125 | 32599734 | Yes | No |
| 125 | 36505186 | Yes | Yes |
| 125 | 45359888 | Yes | No |
| 125 | 50549282 | Yes | Yes |
| 126 | 1979087 | No | Yes |
| 126 | 19303969 | No | Yes |
| 128 | 402605 | Yes | Yes |
| 129 | 2267649 | No | Yes |
| 129 | 2843018 | No | Yes |
| 129 | 3167106 | No | Yes |
| 129 | 3814110 | Yes | Yes |
| 129 | 5528792 | Yes | No |
| 129 | 15497961 | Yes | No |
| 129 | 20290119 | Yes | No |
| 129 | 21076246 | Yes | No |
| 129 | 27462194 | Yes | No |
| 129 | 27789686 | No | Yes |
| 130 | 1385922 | Yes | No |
| 130 | 7313363 | Yes | Yes |
| 130 | 13822684 | No | Yes |
| 130 | 16893610 | Yes | Yes |
| 130 | 19554573 | Yes | Yes |
| 130 | 20253622 | Yes | Yes |
| 130 | 23145427 | Yes | No |
| 130 | 28610106 | Yes | No |
| 130 | 29209130 | Yes | No |
| 130 | 31007551 | No | Yes |
| 130 | 33502362 | Yes | Yes |
| 130 | 37439349 | Yes | Yes |
| 130 | 55021699 | Yes | Yes |
| 130 | 55021752 | Yes | Yes |
| 131 | 24516686 | Yes | No |
| 131 | 32617983 | Yes | No |
| 132 | 759803 | No | Yes |
| 132 | 8484933 | No | Yes |
| 132 | 12881852 | Yes | No |
| 132 | 22703609 | Yes | Yes |
| 132 | 34171303 | Yes | No |
| 133 | 7322903 | No | Yes |
| 133 | 7758805 | Yes | Yes |
| 135 | 3753070 | Yes | Yes |
| 135 | 4710508 | Yes | Yes |
| 135 | 6634747 | Yes | No |
| 136 | 24036 | Yes | No |
| 136 | 4530494 | Yes | No |
| 136 | 5156414 | Yes | Yes |
| 136 | 6061552 | Yes | No |
| 136 | 8672192 | Yes | Yes |
| 136 | 11700532 | Yes | No |
| 136 | 13083451 | No | Yes |
| 136 | 15959760 | No | Yes |
| 136 | 25108138 | Yes | No |
| 136 | 28559105 | Yes | Yes |
| 138 | 2207662 | No | Yes |
| 138 | 6576222 | Yes | No |
| 138 | 7522680 | Yes | No |
| 139 | 2912633 | Yes | Yes |
| 139 | 3237750 | Yes | No |
| 139 | 3819367 | No | Yes |
| 139 | 5780427 | Yes | Yes |
| 139 | 7779949 | Yes | Yes |
| 139 | 8974768 | Yes | Yes |
| 140 | 2346592 | Yes | Yes |
| 140 | 11975037 | Yes | No |
| 140 | 13086103 | Yes | No |
| 140 | 24110031 | Yes | Yes |
| 140 | 26524738 | Yes | No |
| 140 | 28420060 | Yes | No |
| 140 | 31846659 | Yes | Yes |
| 140 | 32702946 | Yes | No |
| 141 | 1744685 | Yes | No |
| 141 | 4399042 | Yes | Yes |
| 141 | 17013124 | Yes | No |
| 142 | 3686644 | Yes | No |
| 144 | 1279354 | Yes | Yes |
| 147 | 3377374 | Yes | Yes |
| 147 | 8909991 | Yes | Yes |
| 147 | 22746581 | Yes | No |
| 147 | 29298916 | No | Yes |
| 148 | 32021 | No | Yes |
| 151 | 282134 | Yes | No |
| 151 | 1098243 | No | Yes |
| 166 | 4208920 | Yes | No |
| 166 | 4288651 | No | Yes |
| 166 | 5809402 | Yes | No |
| 166 | 12163407 | Yes | Yes |
| 166 | 18557803 | No | Yes |
| 183 | 5144436 | No | Yes |
| 183 | 8380627 | No | Yes |
| 183 | 10858518 | Yes | No |
| 185 | 11945908 | Yes | No |
| 221 | 2612425 | No | Yes |
| 222 | 7310415 | No | Yes |
| 222 | 7310512 | Yes | Yes |
| 222 | 8359761 | No | Yes |
| 222 | 10575177 | Yes | Yes |
| 222 | 17339582 | Yes | Yes |
| 222 | 19559951 | Yes | No |
| 222 | 27042142 | Yes | No |
| 222 | 36542945 | No | Yes |
| 222 | 47264341 | No | Yes |
| 222 | 49612080 | Yes | No |
| 222 | 50736492 | Yes | Yes |
| 222 | 54483184 | Yes | Yes |
| 222 | 66754991 | Yes | Yes |
| 222 | 70447108 | Yes | Yes |
| 222 | 73723994 | Yes | Yes |
| 226 | 1164440 | Yes | Yes |
| 244 | 3973341 | No | Yes |
| 244 | 12598565 | Yes | No |
| 244 | 22877206 | No | Yes |
| 259 | 3901551 | Yes | No |
| 259 | 4889569 | Yes | No |
| 259 | 8154569 | Yes | No |
| 259 | 29157243 | Yes | No |
| 259 | 37070332 | Yes | Yes |
| 259 | 41454475 | No | Yes |
| 263 | 4546739 | No | Yes |
| 263 | 9103371 | Yes | Yes |
| 266 | 26386 | Yes | No |
| 266 | 26428 | Yes | No |
| 266 | 1254825 | Yes | No |
| 266 | 3022712 | No | Yes |
| 306 | 2081317 | Yes | No |
| 309 | 16409 | Yes | No |
| 310 | 5026720 | Yes | Yes |
| 310 | 5479433 | Yes | Yes |
| 310 | 12953639 | Yes | Yes |
| 310 | 15007887 | Yes | No |
| 320 | 3957964 | Yes | Yes |
| 320 | 7237786 | Yes | Yes |
| 349 | 2733985 | No | Yes |
| 349 | 8364323 | Yes | Yes |
| 349 | 8364342 | Yes | No |
| 349 | 15835224 | Yes | Yes |
| 349 | 21285993 | Yes | No |
| 349 | 27365013 | Yes | Yes |
| 353 | 637641 | Yes | Yes |
| 353 | 1022770 | No | Yes |
| 386 | 7816521 | Yes | No |
| 386 | 10953446 | Yes | No |
| 397 | 4254 | Yes | No |
| 96520 | 798 | Yes | No |
| 144967 | 3423 | Yes | No |
| 144967 | 3429 | Yes | No |
| 146977 | 2016 | Yes | No |
| 146977 | 2069 | Yes | No |
| 147163 | 32514 | Yes | No |
| 147301 | 219 | Yes | No |
| 309726 | 547 | Yes | No |

Table S5. Standard errors of mean population genomic parameters for the northern quoll (*Dasyurus hallucatus*). Localities have been categorised into treatments according to their history with the presence of cane toads. Parameters include the sample size (n), number of alleles (*A*), effective number of alleles (*A*_E_), SNP expected heterozygosity (*H*_E_), SNP observed heterozygosity (*H*_O_), autosomal expected heterozygosity (*H*_E_ auto), autosomal observed heterozygosity (*H*_O_ auto), Wright’s inbreeding coefficient (*F*_IS_), and the locus polymorphic index (*P*_E_). Localities have been ordered by treatment rather than geographic location.

| Locality | Treatment | Region | n | *A* | *A*_E_ | *H*_E_ (SNP) | *H*_O_ (SNP) | *H*_E_ (auto × 100) | *H*_O_ (auto × 100) | *F*_IS_ | *P*_E_ |
| --- | --- | --- | --- | --- | --- | --- | --- | --- | --- | --- | --- |
| Darwin | Naïve | NT | 9 | 0.004 | 0.002 | 0.001 | 0.001 | 0.000 | 0.000 | 0.007 | 0.001 |
| Outer Darwin | Naïve | NT | 49 | 0.005 | 0.002 | 0.001 | 0.001 | 0.000 | 0.000 | 0.004 | 0.001 |
| East Alligator (2003) | Naïve | NT | 14 | 0.004 | 0.002 | 0.001 | 0.001 | 0.000 | 0.000 | 0.007 | 0.001 |
| Kapalga | Naïve | NT | 21 | 0.004 | 0.002 | 0.001 | 0.001 | 0.000 | 0.000 | 0.005 | 0.001 |
| Artesian Range | Naïve | WA | 19 | 0.005 | 0.002 | 0.001 | 0.001 | 0.000 | 0.000 | 0.004 | 0.001 |
| Bachsten Creek | Naïve | WA | 9 | 0.004 | 0.002 | 0.001 | 0.001 | 0.000 | 0.000 | 0.005 | 0.001 |
| Wunaamin Miliwundi E | Naïve | WA | 11 | 0.004 | 0.002 | 0.001 | 0.001 | 0.000 | 0.000 | 0.005 | 0.001 |
| Wunaamin Miliwundi W | Naïve | WA | 12 | 0.004 | 0.002 | 0.001 | 0.001 | 0.000 | 0.000 | 0.005 | 0.001 |
| Millstream Chichester NP | Naïve | WA | 8 | 0.003 | 0.002 | 0.001 | 0.001 | 0.000 | 0.000 | 0.008 | 0.001 |
| Mornington Sanctuary | Naïve | WA | 15 | 0.004 | 0.002 | 0.001 | 0.001 | 0.000 | 0.000 | 0.006 | 0.001 |
| Marble Bar | Naïve | WA | 8 | 0.003 | 0.002 | 0.001 | 0.001 | 0.000 | 0.000 | 0.008 | 0.001 |
| Black Mountain | Exposed | QLD | 6 | 0.002 | 0.001 | 0.001 | 0.001 | 0.000 | 0.000 | 0.012 | 0.001 |
| East Alligator (2014) | Exposed | NT | 6 | 0.003 | 0.002 | 0.001 | 0.001 | 0.000 | 0.000 | 0.009 | 0.001 |
| East Alligator (2017) | Exposed | NT | 8 | 0.003 | 0.002 | 0.001 | 0.001 | 0.000 | 0.000 | 0.009 | 0.001 |
| Hope Vale | Exposed | QLD | 6 | 0.002 | 0.001 | 0.001 | 0.001 | 0.000 | 0.000 | 0.011 | 0.001 |
| Mareeba | Exposed | QLD | 8 | 0.003 | 0.001 | 0.001 | 0.001 | 0.000 | 0.000 | 0.011 | 0.001 |
| Weipa | Exposed | QLD | 12 | 0.002 | 0.001 | 0.001 | 0.001 | 0.000 | 0.000 | 0.018 | 0.001 |
| Groote West | Island | NT | 16 | 0.003 | 0.002 | 0.001 | 0.001 | 0.000 | 0.000 | 0.013 | 0.001 |
| Koolan Island | Island | WA | 9 | 0.003 | 0.002 | 0.001 | 0.001 | 0.000 | 0.000 | 0.011 | 0.001 |
| Marchinbar Island | Island | NT | 26 | 0.001 | 0.000 | 0.000 | 0.000 | 0.000 | 0.000 | 0.058 | 0.000 |
| Astell Island (2005) | Translocated | NT | 17 | 0.004 | 0.002 | 0.001 | 0.001 | 0.000 | 0.000 | 0.006 | 0.001 |
| Astell Island (2016) | Translocated | NT | 13 | 0.004 | 0.002 | 0.001 | 0.001 | 0.000 | 0.000 | 0.007 | 0.001 |
| Pobassoo Island | Translocated | NT | 15 | 0.004 | 0.002 | 0.001 | 0.001 | 0.000 | 0.000 | 0.007 | 0.001 |

Table S6. Standard deviations of mean population genomic parameters for the northern quoll (*Dasyurus hallucatus*). Localities have been categorised into treatments according to their history with the presence of cane toads. Parameters include the sample size (n), number of alleles (*A*), effective number of alleles (*A*_E_), SNP expected heterozygosity (*H*_E_), SNP observed heterozygosity (*H*_O_), autosomal expected heterozygosity (*H*_E_ auto), autosomal observed heterozygosity (*H*_O_ auto), Wright’s inbreeding coefficient (*F*_IS_), and the locus polymorphic index (*P*_E_). Localities have been ordered by treatment rather than geographic location.

| Locality | Treatment | Region | n | *A* | *A*_E_ | *H*_E_ (SNP) | *H*_O_ (SNP) | *H*_E_ (auto × 100) | *H*_O_ (auto × 100) | *F*_IS_ | *P*_E_ |
| --- | --- | --- | --- | --- | --- | --- | --- | --- | --- | --- | --- |
| Darwin | Naïve | NT | 9 | 0.381 | 0.186 | 0.117 | 0.122 | 0.004 | 0.004 | 0.286 | 0.110 |
| Outer Darwin | Naïve | NT | 49 | 0.458 | 0.182 | 0.108 | 0.105 | 0.003 | 0.004 | 0.230 | 0.107 |
| East Alligator (2003) | Naïve | NT | 14 | 0.385 | 0.187 | 0.114 | 0.116 | 0.004 | 0.004 | 0.281 | 0.109 |
| Kapalga | Naïve | NT | 21 | 0.442 | 0.178 | 0.109 | 0.105 | 0.004 | 0.004 | 0.264 | 0.106 |
| Artesian Range | Naïve | WA | 19 | 0.463 | 0.193 | 0.117 | 0.119 | 0.005 | 0.005 | 0.208 | 0.114 |
| Bachsten Creek | Naïve | WA | 9 | 0.421 | 0.197 | 0.124 | 0.132 | 0.005 | 0.006 | 0.247 | 0.116 |
| Wunaamin Miliwundi E | Naïve | WA | 11 | 0.417 | 0.200 | 0.123 | 0.128 | 0.005 | 0.005 | 0.242 | 0.118 |
| Wunaamin Miliwundi W | Naïve | WA | 12 | 0.431 | 0.196 | 0.121 | 0.126 | 0.005 | 0.005 | 0.229 | 0.116 |
| Millstream Chichester NP | Naïve | WA | 8 | 0.344 | 0.201 | 0.123 | 0.134 | 0.005 | 0.005 | 0.285 | 0.114 |
| Mornington Sanctuary | Naïve | WA | 15 | 0.374 | 0.208 | 0.123 | 0.126 | 0.005 | 0.005 | 0.255 | 0.118 |
| Marble Bar | Naïve | WA | 8 | 0.336 | 0.198 | 0.121 | 0.135 | 0.005 | 0.005 | 0.303 | 0.113 |
| Black Mountain | Exposed | QLD | 6 | 0.239 | 0.130 | 0.084 | 0.089 | 0.004 | 0.004 | 0.295 | 0.076 |
| East Alligator (2014) | Exposed | NT | 6 | 0.347 | 0.196 | 0.124 | 0.124 | 0.004 | 0.004 | 0.345 | 0.113 |
| East Alligator (2017) | Exposed | NT | 8 | 0.335 | 0.191 | 0.118 | 0.126 | 0.004 | 0.004 | 0.331 | 0.110 |
| Hope Vale | Exposed | QLD | 6 | 0.247 | 0.133 | 0.085 | 0.091 | 0.004 | 0.005 | 0.283 | 0.078 |
| Mareeba | Exposed | QLD | 8 | 0.263 | 0.136 | 0.086 | 0.089 | 0.004 | 0.005 | 0.292 | 0.080 |
| Weipa | Exposed | QLD | 12 | 0.181 | 0.113 | 0.067 | 0.075 | 0.003 | 0.004 | 0.340 | 0.064 |
| Groote West | Island | NT | 16 | 0.272 | 0.166 | 0.097 | 0.096 | 0.004 | 0.004 | 0.382 | 0.094 |
| Koolan Island | Island | WA | 9 | 0.271 | 0.182 | 0.107 | 0.113 | 0.004 | 0.004 | 0.321 | 0.101 |
| Marchinbar Island | Island | NT | 26 | 0.075 | 0.043 | 0.024 | 0.032 | 0.000 | 0.000 | 0.432 | 0.023 |
| Astell Island (2005) | Translocated | NT | 17 | 0.388 | 0.187 | 0.113 | 0.118 | 0.004 | 0.004 | 0.266 | 0.109 |
| Astell Island (2016) | Translocated | NT | 13 | 0.386 | 0.187 | 0.115 | 0.116 | 0.004 | 0.004 | 0.296 | 0.110 |
| Pobassoo Island | Translocated | NT | 15 | 0.363 | 0.187 | 0.113 | 0.117 | 0.004 | 0.004 | 0.290 | 0.109 |

Table S7. Population genomic parameters for the northern quoll (*Dasyurus hallucatus*). Values represent means across sampling localities, where each locality was categorised into one of four treatments according to their history with the presence of cane toads. Parameters include the number of alleles (*A*), effective number of alleles (*A*_E_), SNP expected heterozygosity (*H*_E_), SNP observed heterozygosity (*H*_O_), autosomal expected heterozygosity (*H*_E_ auto), autosomal observed heterozygosity (*H*_O_ auto), Wright’s inbreeding coefficient (*F*_IS_), and the locus polymorphic index (*P*_E_).

| Treatment | *A* | *A*_E_ | *H*_E_ (SNP) | *H*_O_ (SNP) | *H*_E_ (auto × 100) | *H*_O_ (auto × 100) | *F*_IS_ | *P*_E_ |
| --- | --- | --- | --- | --- | --- | --- | --- | --- |
| Toad-exposed | 1.084 | 1.039 | 0.027 | 0.026 | 0.005 | 0.006 | 0.012 | 0.024 |
| Island | 1.055 | 1.030 | 0.019 | 0.018 | 0.003 | 0.003 | 0.020 | 0.018 |
| Toad-naïve | 1.215 | 1.072 | 0.049 | 0.048 | 0.009 | 0.009 | 0.014 | 0.047 |
| Translocated | 1.174 | 1.064 | 0.043 | 0.042 | 0.006 | 0.007 | 0.023 | 0.042 |


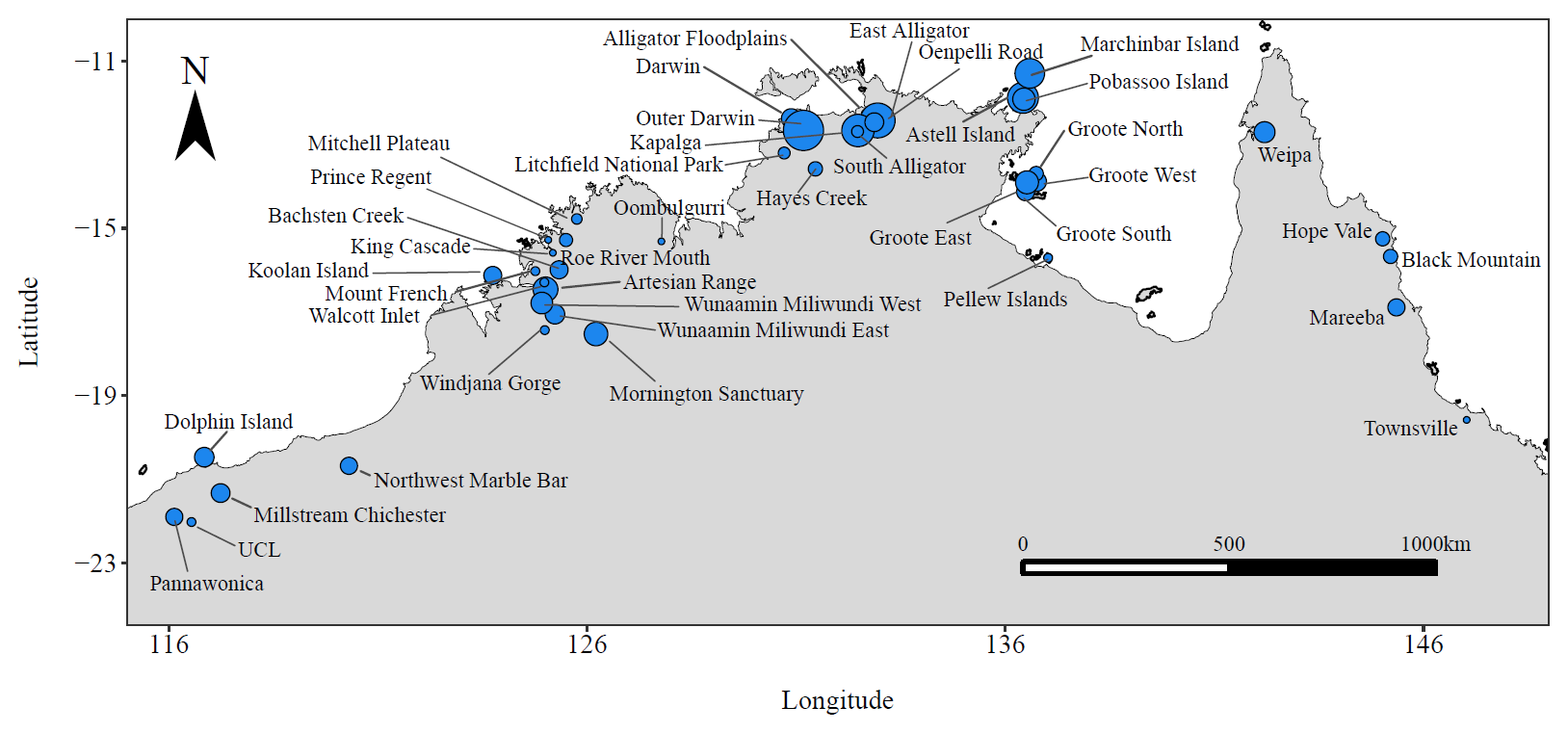


Figure S1. Map showing location of all northern quoll populations sampled for DNA extraction. Points are sized by the number of tissue samples collected.

Figure S2. Hierarchical clustering dendrogram representing genetic distance relationships between *Dasyurus hallucatus* samples. Calculations were made using 10,676 single-nucleotide polymorphisms across the genome. Technical replicates are identified with an ‘x’ for within-plate replicates, or ‘xx’ for among-plate replicates, after the sample name and are paired together on branches.


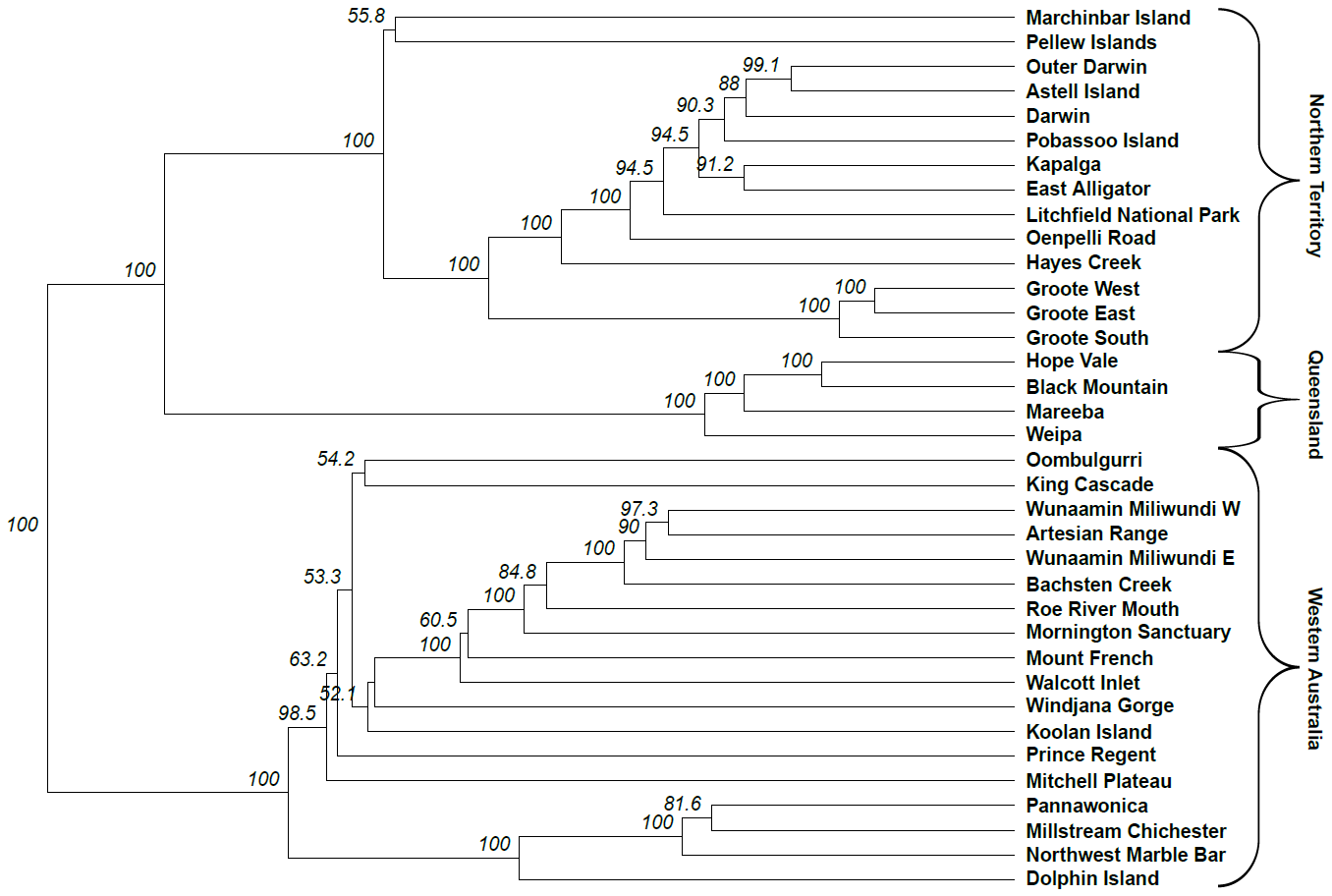


Figure S3. Hierarchical clustering dendrogram visualising genetic distance between *Dasyurus hallucatus* populations. Calculations were made using 10,676 single-nucleotide polymorphisms across the genome, with values on nodes representing the level of bootstrap support based on 1000 random samples of loci.


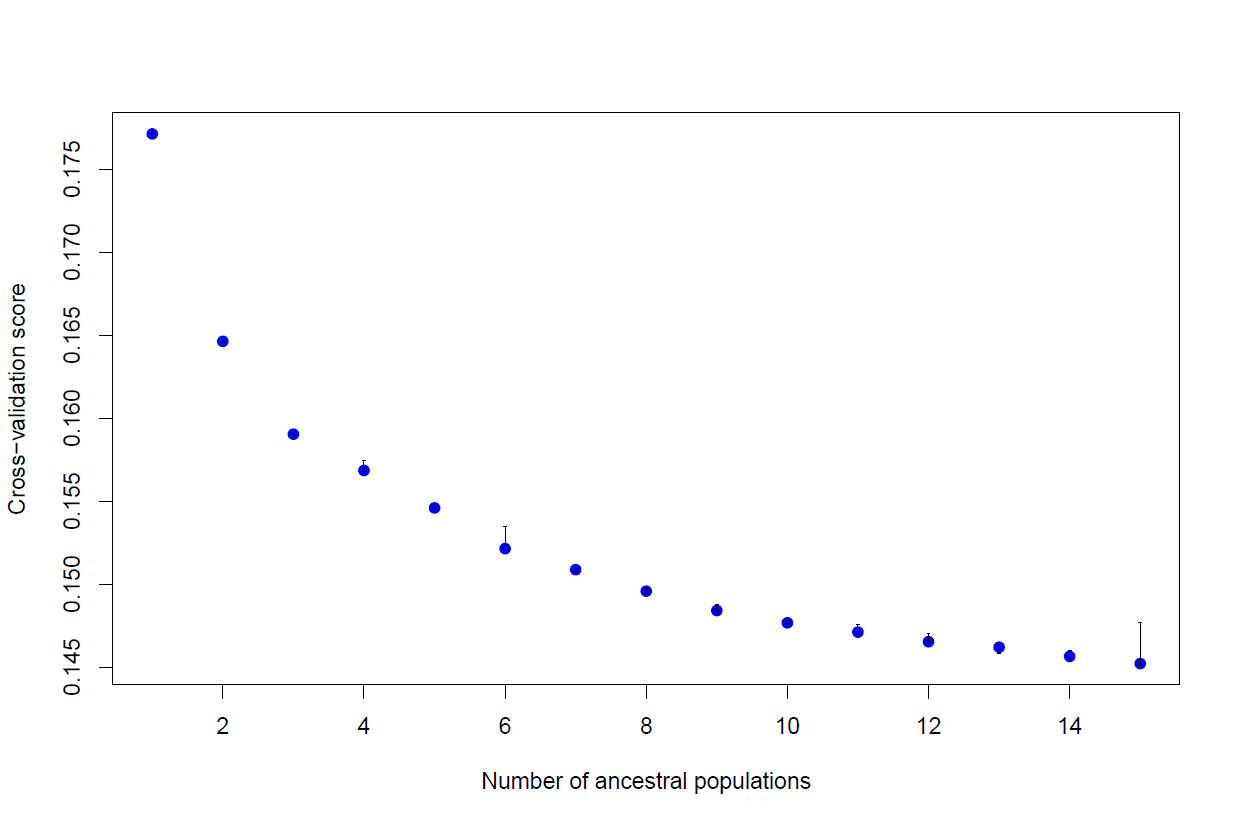


Figure S4. Cross-entropy plot used to identify hierarchical population structuring in the genomic dataset for northern quolls (*Dasyurus hallucatus*). Lower values of the cross-entropy criterion indicate a better fit to the data. The large drops in cross-entry between 1 and 2, and 2 and 3 ancestral populations indicates that *k* values of 2 or 3 are well-supported, although higher levels of *k* further subdivide *a-priori* populations into clusters that correspond with the spatial pattern of sampling.


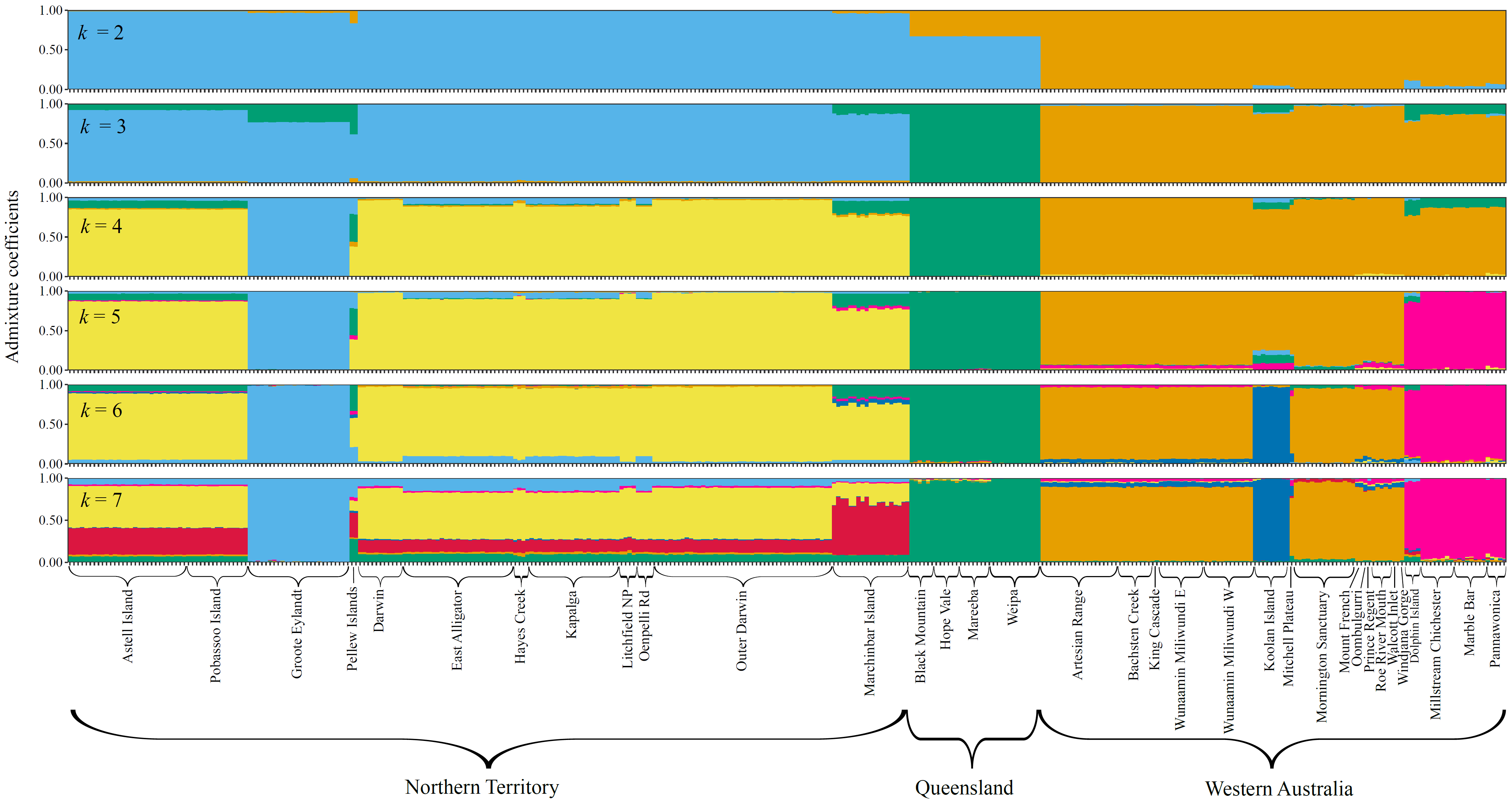


Figure S5. Patterns of population genomic structure in the northern quoll. Panels show the structure when the number of ancestral genomic clusters (*k*) is increased from two to five. Each individual is represented by a vertical bar, coloured by membership proportion to ancestral clusters. Colours match those shown in Figure 3 of the main document.


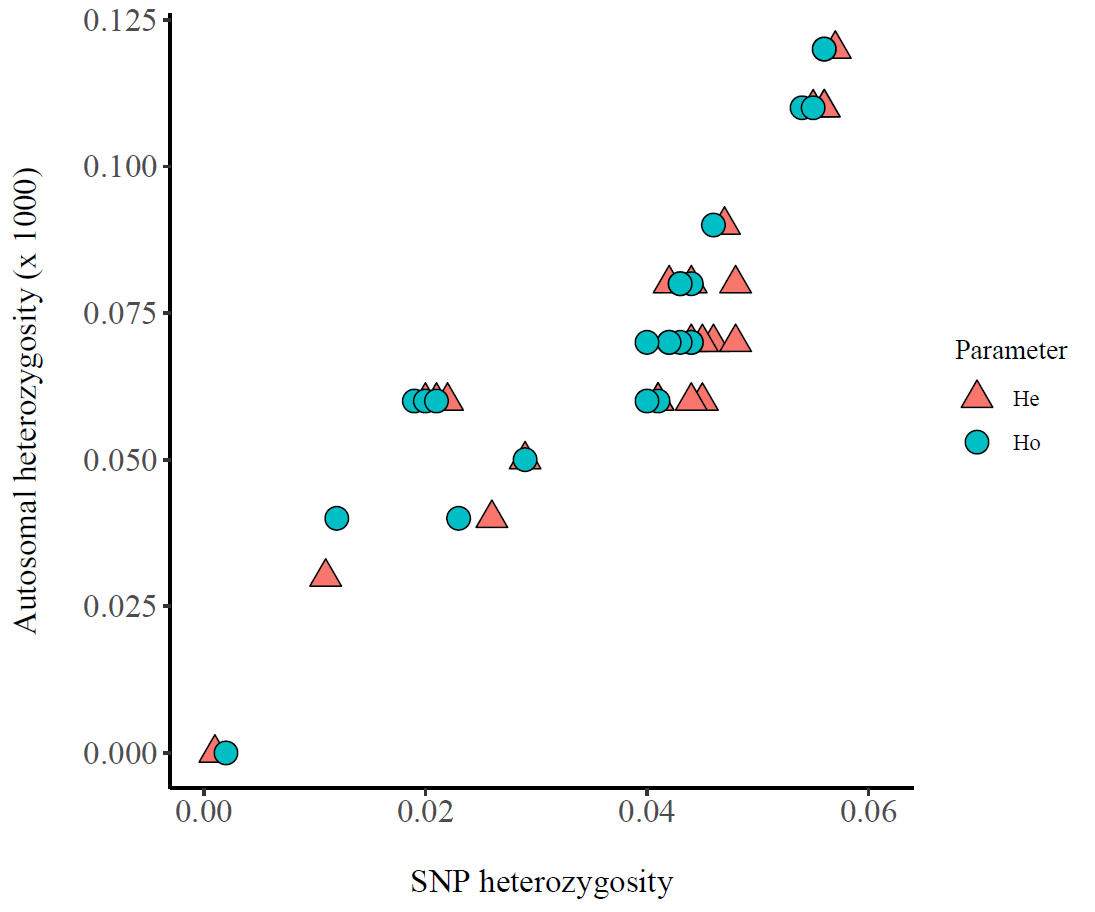


Figure S6. Plot of filtered SNP heterozygosity against autosomal heterozygosity for populations of northern quolls where *n* ≥ 6. Both observed and expected heterozygosity values are shown. SNP heterozygosity was calculated using 10,191 SNPs after stringent filtering, whereas autosomal heterozygosity was calculated based on monomorphic and polymorphic sites, where 55,970 sites from a total of 18,879,074 sites were variable.


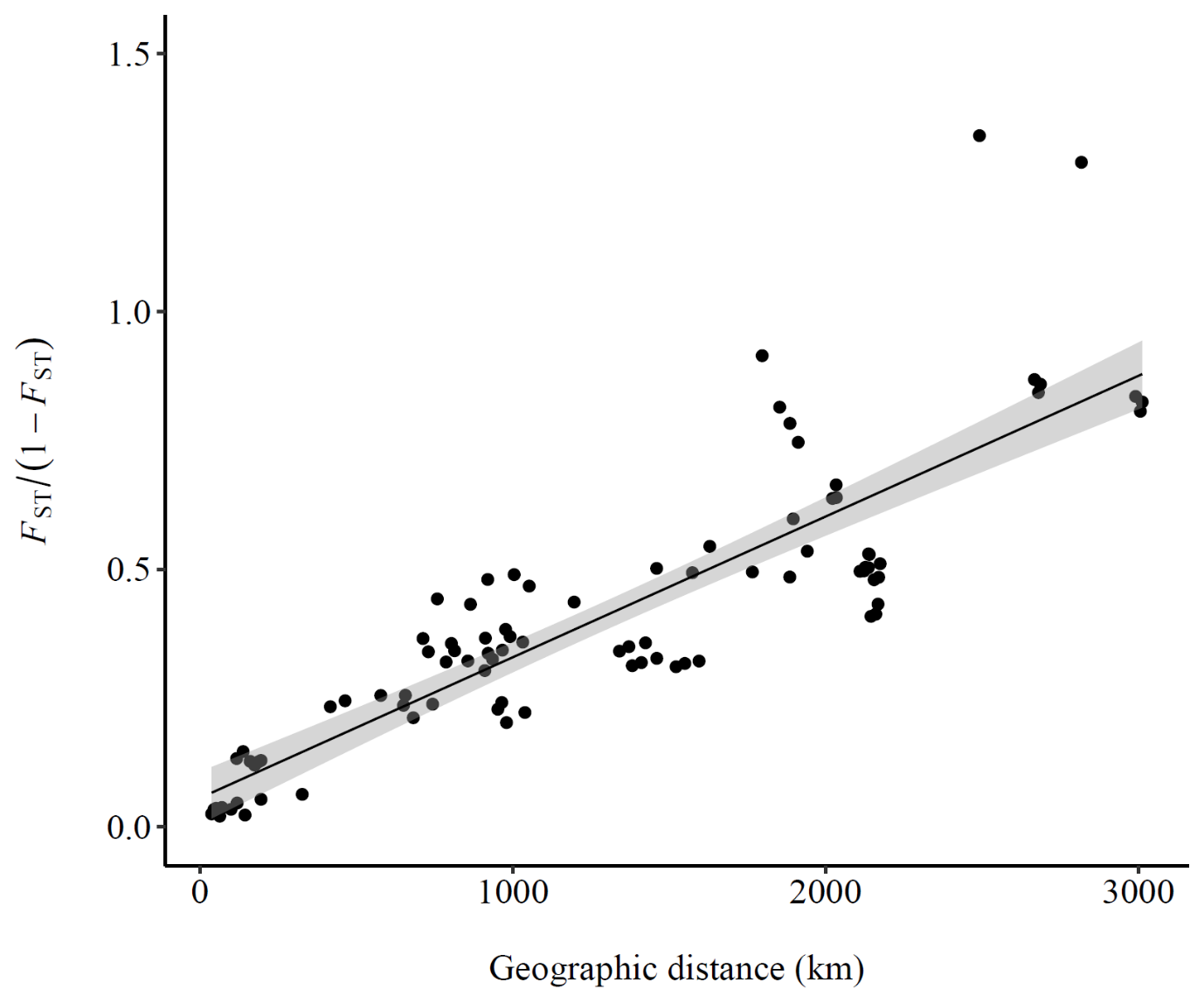


Figure S7. Pattern of (standardised) genetic differentiation by geographic distance in northern quolls for sampled localities (n ≥ 6) from across the mainland distribution of the species. Island populations have been removed.


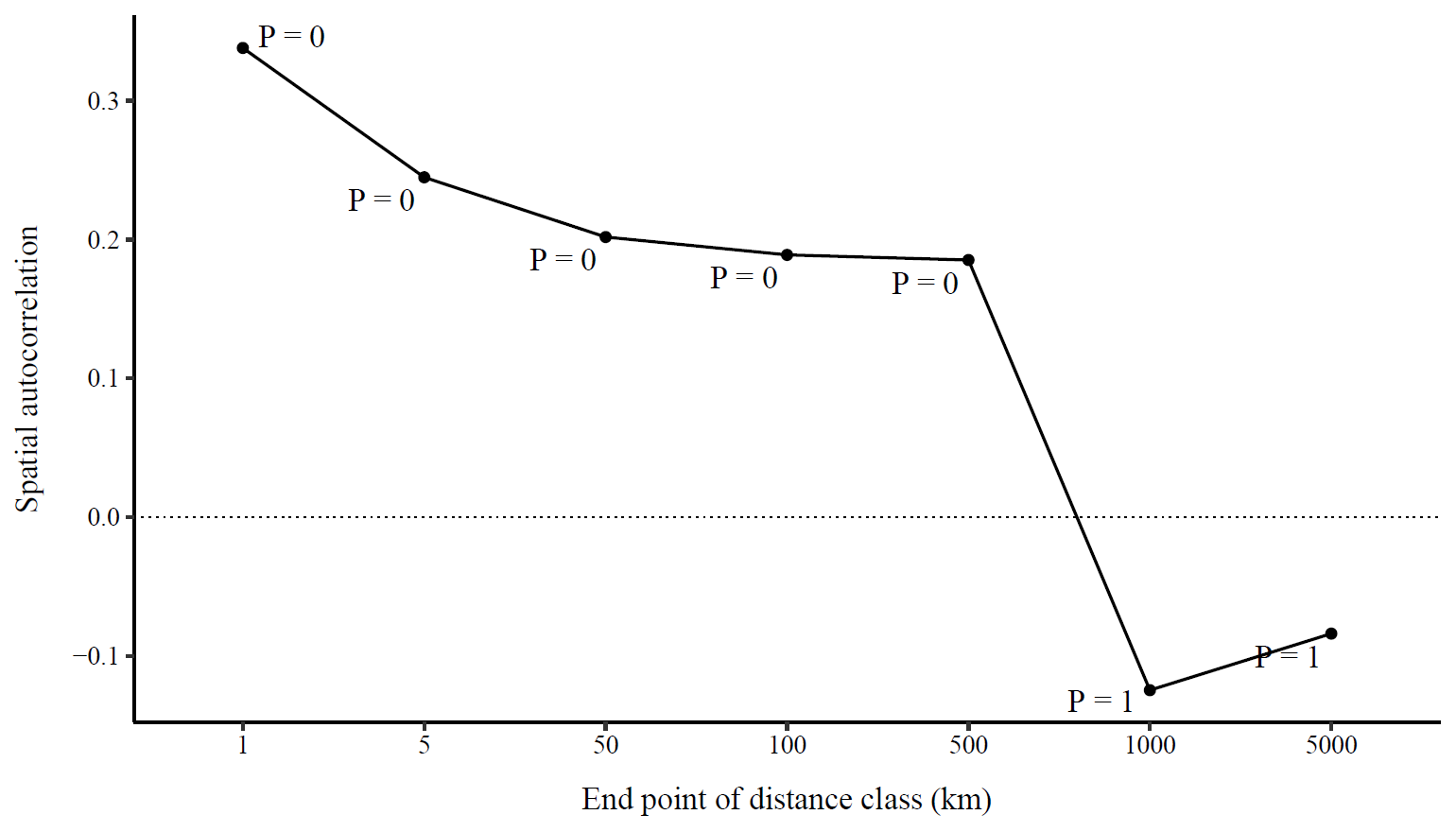


Figure S8. Spatial autocorrelation of multilocus genotypes for individual northern quolls (*Dasyurus hallucatus*) on mainland Australia at seven distance classes. The probability value at each distance class shows the proportion of permuted *r* values greater than the observed value in that distance class, based on 999 permutations of the SNP by sample matrix.


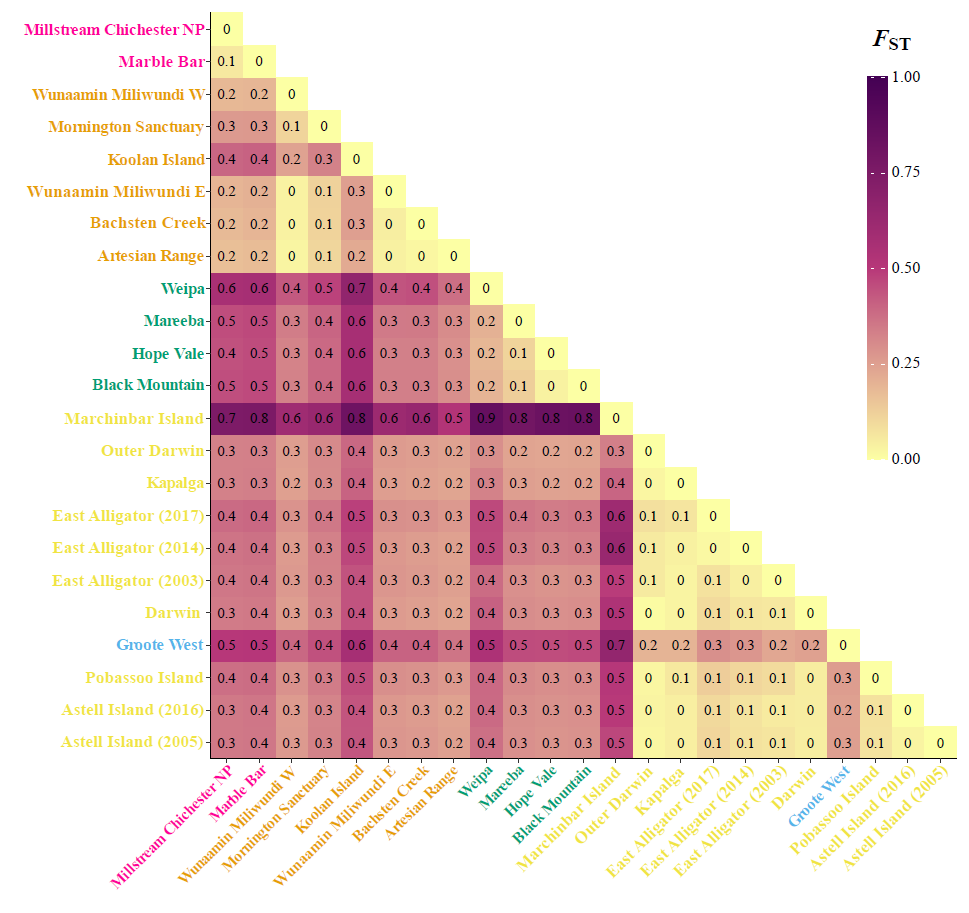


Figure S9. Pairwise genomic differentiation (*F*_ST_) values between populations of the northern quoll (where n ≥ 8). All *F*_ST_ values are significant at *p* < 0.001. Localities are labelled with colours matching those used in Figure 3 (where *k* = 5).


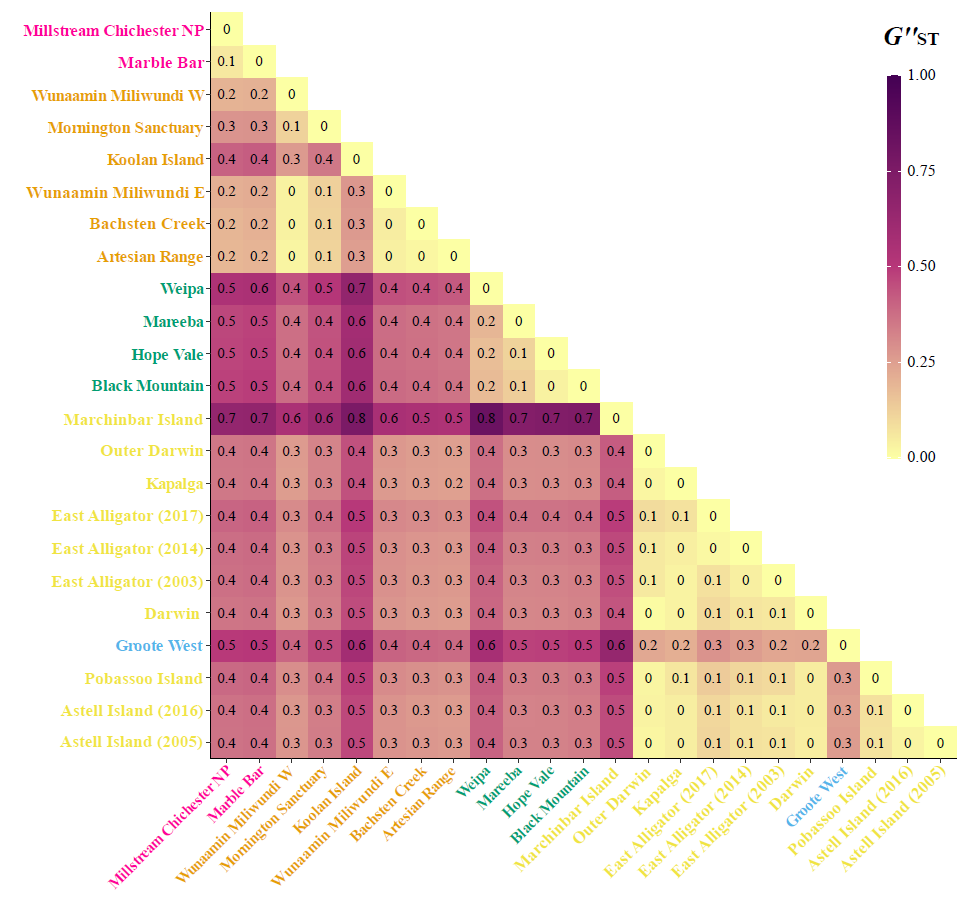


Figure S10. Pairwise genomic differentiation (*G*ꞌꞌ_ST_) values between populations of the northern quoll (where n ≥ 8). Localities are labelled with colours matching those used in Figure 3 (*k* = 5).


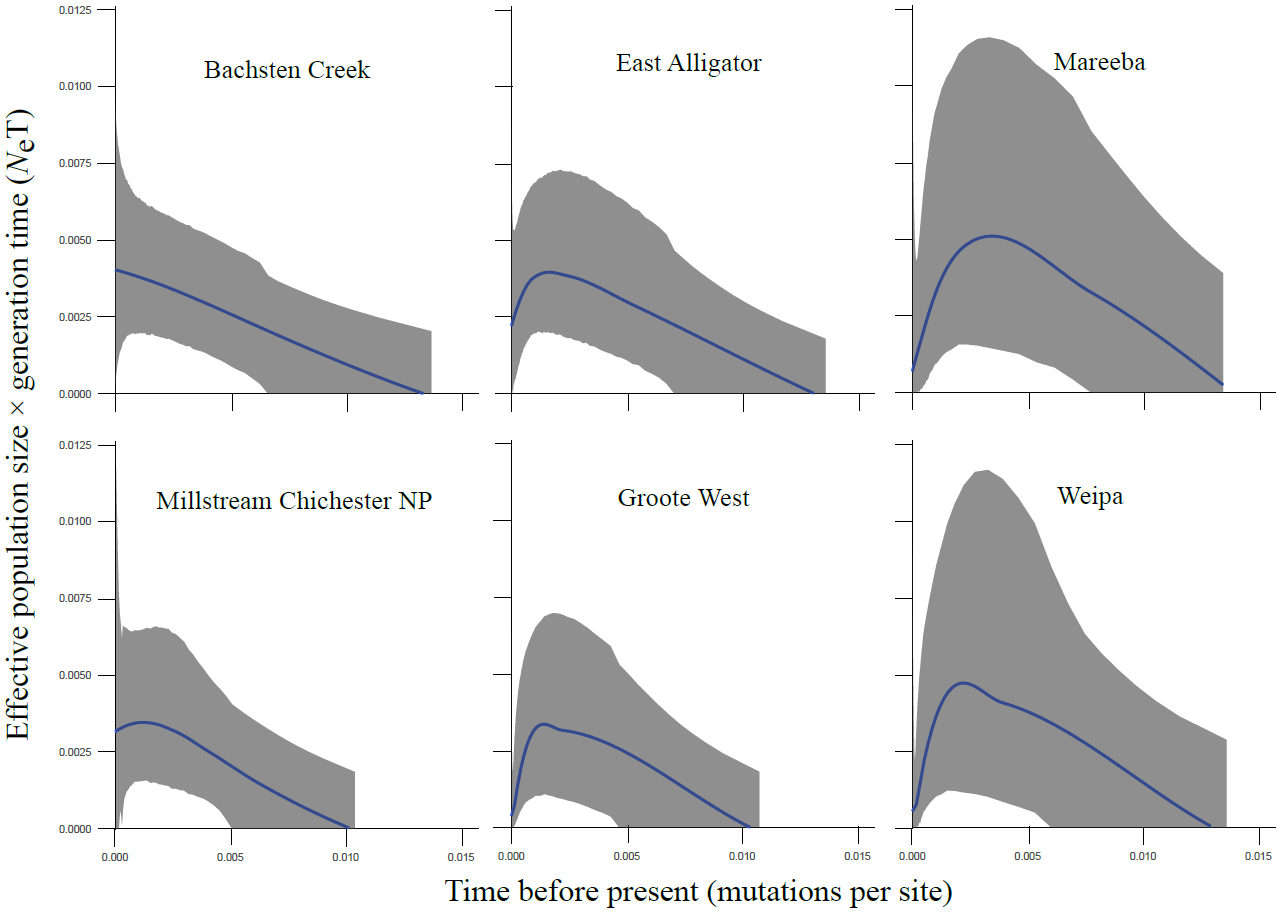


Figure S11. Extended Bayesian skyline plots illustrating the posterior distribution of demographic trends for six northern quoll populations. The median effective population size (*N*_e_) is shown by the blue line, with 95% high posterior density shown in grey. Populations on the right-hand side (Mareeba and Weipa) have been exposed to cane toads whereas other populations have not.
